# Upconversion optogenetic micro-nanosystem optically controls the secretion of light-responsive bacteria for systemic immunity regulation

**DOI:** 10.1038/s42003-020-01287-4

**Published:** 2020-10-09

**Authors:** Chun Yang, Meihui Cui, Yingying Zhang, Huizhuo Pan, Jing Liu, Shixing Wang, Ning Ma, Jin Chang, Tao Sun, Hanjie Wang

**Affiliations:** 1 School of Life Sciences, Tianjin University, Tianjin, 300072 China; 2 Tianjin Engineered Center of Micro-Nano Biomaterials and Detection-Treatment Technology, Tianjin Key Laboratory of Function and Application of Biological Macromolecular Structures, Tianjin, 300072 China; 3 Academy of Medical engineered and Translational Medicine, Tianjin University, Tianjin, 300072 China; 4 Center for Biosafety Research and Strategy, Tianjin University, Tianjin, 300072 China; 5 Laboratory of Synthetic Microbiology, School of Chemical engineered and Technology, Tianjin University, Tianjin, 300072 China

**Keywords:** Applied microbiology, Ulcerative colitis

## Abstract

Chemical molecules specifically secreted into the blood and targeted tissues by intestinal microbiota can effectively affect the associated functions of the intestine especially immunity, representing a new strategy for immune-related diseases. However, proper ways of regulating the secretion metabolism of specific strains still remain to be established. In this article, an upconversion optogenetic micro-nanosystem was constructed to effectively regulate the specific secretion of engineered bacteria. The system included two major modules: (i) Modification of secretory light-responsive engineered bacteria. (ii) Optical sensing mediated by upconversion optogenetic micro-nanosystem. This system could regulate the efficient secretion of immune factors by engineered bacteria through optical manipulation. Inflammatory bowel disease and subcutaneously transplanted tumors were selected to verify the effectiveness of the system. Our results showed that the endogenous factor TGF-β1 could be controllably secreted to suppress the intestinal inflammatory response. Additionally, regulatory secretion of IFN-γ was promoted to slow the progression of B16F10 tumor.

## Abstract (web summary)

Yang, Cui, Zhang et al. report an optogenetic nanosystem which controls the secretion of immune factors by engineered light-responsive bacteria for systemic immunity regulation. On shining NIR light on mice’s stomach, blue light emission from upconversion rods induce excretion of TGF-β1 and IFN-*γ* from the bacteria, thereby suppressing intestinal inflammatory response or slowing down progress of subcutaneously transplanted tumour, respectively.

## Introduction

Intestinal microbiota is an extremely complex group, which is considered as the “second genome” of human organs or human beings due to its important role^1^. Different numbers and types of bacteria are colonized in different parts of the human intestinal tract. They play important roles in maintaining the normal structure and physiological function of intestinal tract, antagonizing colonization, infection, and stimulation of pathogenic microorganisms, as well as affecting physiological functions of the body^2^. These functions are interrelated and mutually reinforcing, thus maintaining the balance of the body. On the other hand, as the body’s largest mucosal system, the intestine also has extremely rich blood flow, lymphatic drainage, and a sophisticated regional immune system. The intestinal immune system and intestinal microbiota are located on both sides of the mucosal barrier, affecting each other in a variety of ways, and may affect the body’s immune and physiological processes.

The most important function of the human immune system is to recognize and respond to many allogeneic and autoimmune active components. As a large number of heterogeneous components, the intestinal microbiota has a complex two-way effect on the immune system. In short, the symbiosis of the microbiota and the human body depends on the formation of immune tolerance^3^. The development of immune tissues and the activation of immune cells are also closely affected by the microbiota signal^4^. The key to this vector signal is the type of microbiota secretion. An immune-active microbiota secretion can directly guide the response to downstream immune signals. Notably, attempts have been made to use microbiota-specific secretions to influence the immune status during disease treatment^5^. Clinical studies have shown that probiotic supplementation and fecal microbiota transplantation (FMT) have been used in combination with traditional immunotherapy^6,7^. However, the above approaches still fail to achieve efficient regulation of specific strain secretions. The interaction of microbiota secretions alone cannot control the immune process, which is difficult to effectively control the improvement of the immune environment. Although an exogenous chemical inducer might be used to regulate the secretion of engineered strains, delayed expression and unpredictable toxic side effects could be caused. Therefore, it is particularly important how to effectively design and utilize the secretion process of specific strains to regulate the specific immune metabolites and finally improve the systemic immune environment.

The emergence of optogenetics provides an important strategy for the regulation of expression of specific strains. Optogenetics is a rapid development in recent years, a cross-cutting bioengineered technology that integrates optical, software control, gene operation technology, and electrophysiology, etc.^8,9^. It genetically encodes a light-sensitive protein of which the structure changes under light irradiation to control the physiological state of cells in temporal and spatial dimensions. Its non-invasive regulatory model overcomes many shortcomings of traditional methods in controlling the cell or biological activity^10–12^, achieving a wide range of applications in neuroscience, medical engineering, and other fields. In this study, we set up an upconversion optogenetic Micro-nanosystem. The core link was the construction of light-responsive engineered bacteria plasmids using genetic manipulation techniques. This plasmid was used as a genetic vector for the directional construction of optogenetic engineered strains, which can effectively secrete immunoactive molecules by optical manipulation. Compared with traditional microbiota transplantation methods (including probiotics and FMT), this regulation model had the advantages of high efficiency and sensitivity for the secretion regulation of engineered bacteria and the reduced toxic side effects. In practical applications, upconversion rods (UCRs; a kind of rare-earth-doped materials) were also introduced to improve the efficiency of optical signal transmission and reduced signal loss caused by biological tissue penetration^13,14^. Upconversion materials had a strong photoluminescence characteristic and could emit visible light with different wavelengths when rare-earth elements in various ratio were doped to achieve the manipulation of optogenetics. In summary, the whole system includes two major modules as follows: (i) construction of optical-responsive engineered strains and (ii) optical sensing mediated by UCRs. It can realize the light response of engineered bacteria to secrete immunoactive molecules.

**Fig. 1:**
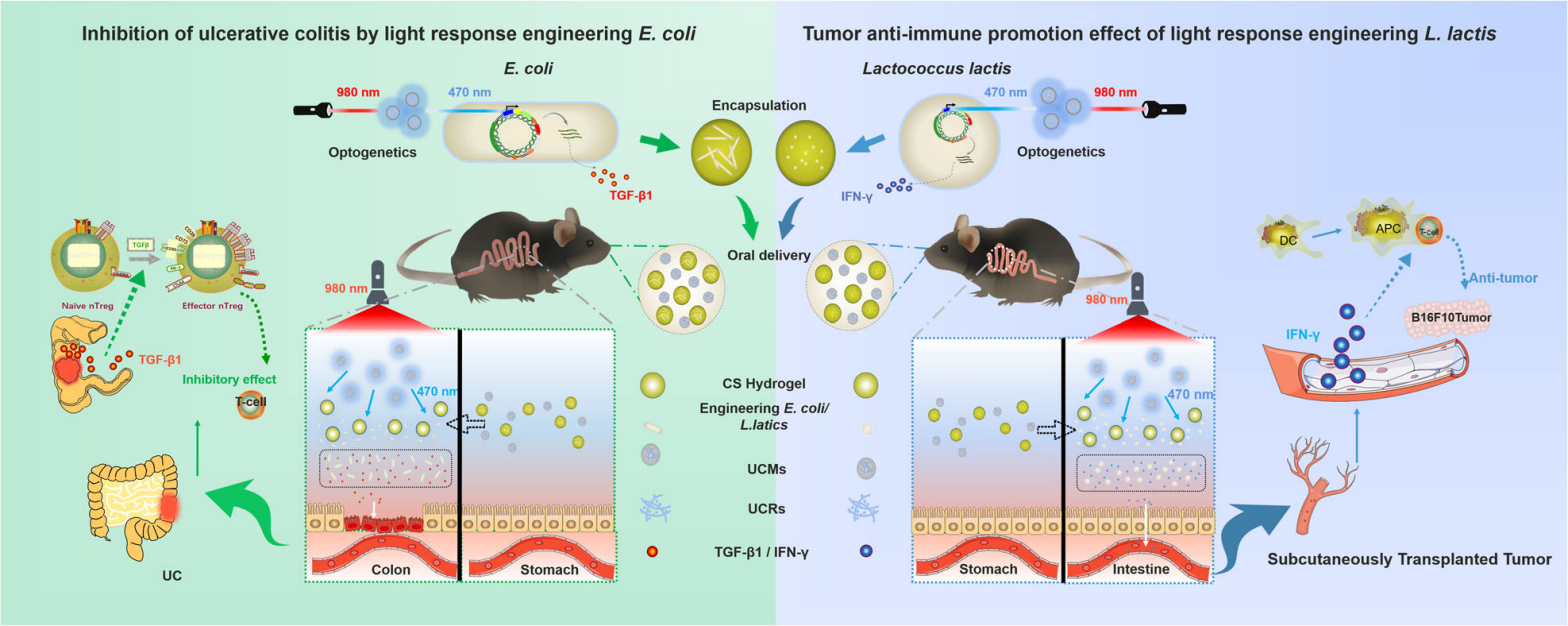
Schematic diagram of the upconversion optogenetic micro-nanosystem. Construction, packaging, delivery and optical application of light-responsive engineering strains. Two application scenarios: inhibition of ulcerative colitis, and tumor anti-immune promotion effect.

To verify the feasibility of the regulation mode, two representative types of immune-related diseases (i.e., inflammatory bowel disease and subcutaneous transplanted tumor) were used as examples, respectively, to verify its immune effect. Ulcerative colitis (UC) is a chronic disorder causing prolonged inflammation of the gastrointestinal tract^15^. Throughout the entire process of the initiation, progression, and severity of UC, there are complex molecular interactions and cellular communications among different proinflammatory cytokines/chemokines, oxidative mediators, inflammatory cells, and immune cells. The inflammatory molecules, immune cells, and microbial community lead to the proinflammatory microenvironment in the intestine that causes UC^16,17^. Consequently, immune-active substances secreted by the microbiota are interesting and are effective strategies to restore the abnormal inflammatory environment of UC. Besides, tumor immunotherapy is another hot topic, which can kill cancer cells by activating the body’s own immune system. This requires the collaborative participation of related immune cells and organs to constantly improve the malignant tumor environment^18^. With the further development of tumor immunotherapy, the improvement of its immune microenvironment has become an important key point. If the systemic immune system can be effectively enhanced through the continuous secretion of immunologically active molecules, it can be helpful to achieve efficient implementation of tumor therapy^19^. As an immune defense system with systemic characteristics, the far-reaching effects of immune molecules on their effects through the metabolic cycle of the body will also become a significant concern^20^. Based on the above two immune-related diseases, we will discuss the significance of the “light-responsive strain-immunity” regulatory model and the possibility of guiding the prevention and remission of related diseases (Fig. 1).

## Results

### Blue light-induced downstream gene expression

**Fig. 2:**
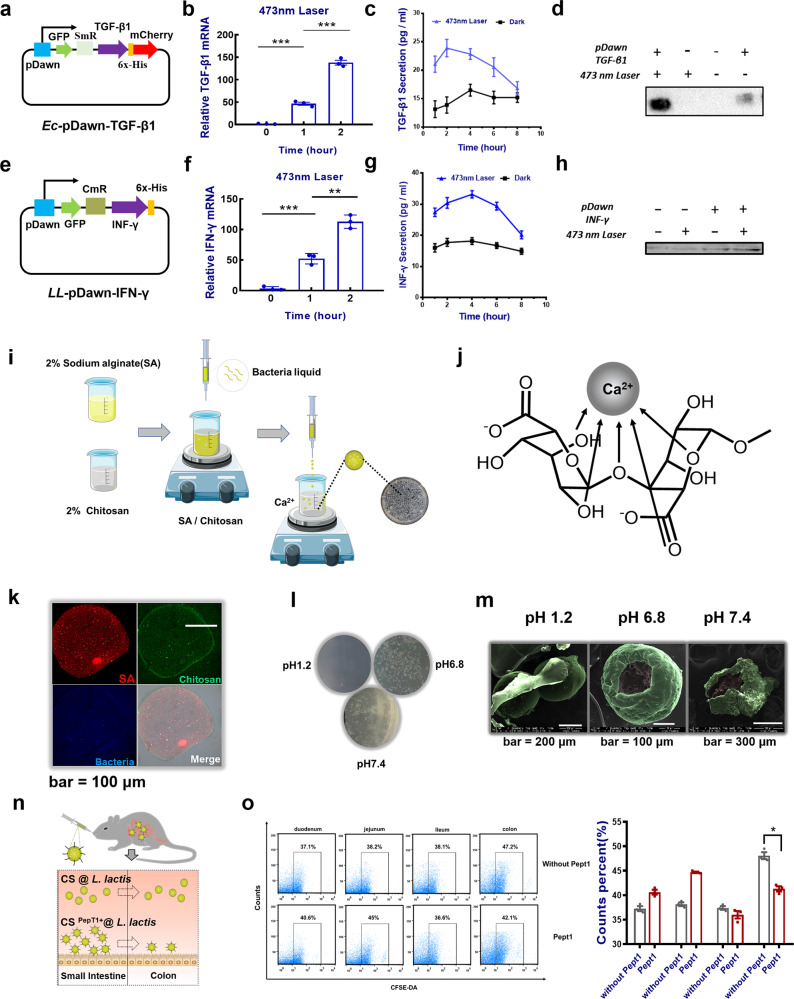
Modification of secretory light-responsive engineered bacteria. **a** Schematic diagram of light-responsive plasmid pDawn. **b** Construction of pDawn-GFP engineered bacteria. **c** The growth of light-responsive engineered bacteria in dark or blue light. **d** Flow cytometry detection of GFP expression with blue light irradiation. **e** GFP expression of engineered bacteria induced by blue light under an inverted fluorescence microscope. **f** Growth curves of *E. coli* under 473 nm blue laser with different power intensity (OD600 absorbance). **g** Growth curves of *L. lactis* under 473 nm blue laser with different power intensity (OD600 absorbance). **h** MTT cell viability assay of 473 nm blue light (*n* = 5). Data are expressed as the mean ± SEM.

The greatest advantage of the above system is the high precision and high efficiency. The key to its operation lies in the efficiency of optical signal response and the gene expression effect of downstream destination. Obviously, the first link is the construction of light-sensitive engineered plasmid^21,22^. It converts light signals such as electromagnetic waves, into intracellular signals that regulate the expression of the target product. This requires engineered plasmids to have two modules that perform the main functions. The first is the sensor module that absorbs light and the second is the effector module that performs biological activity. In previous studies, Ohlendorf et al.^23^ constructed a single plasmid system pDawn based on the YFl/FiXJ system, which could efficiently initiate the expression of subsequent genes once absorbing blue light. In the absence of blue light, YFl will phosphorylate the same response regulator, FixJ, which is phosphated to drive gene expression after the FixK2 promoter. In the presence of 473 nm blue light (20 Hz), the net kinase activity of YFl and the subsequent gene expression are reduced (Fig. 2a). To verify the light response efficiency of the pDawn plasmid system, the green fluorescent protein (GFP) gene was introduced to downstream fragments as a reporter gene. The validation plasmid was transferred to the *Escherichia coli* BL21(Ec-pDawn-GFP) competent cell for blue light induction at different time periods (Fig. 2b). The fluorescence microscope images and flow cytometry analysis showed that the apparent expression of GFP could be observed within 30 min of exposure to blue light by *E. coli* in the arithmetic (~OD_600_ = 0.5) (Fig. 2d, e). After raising the blue light induction time to 10 h, the same amount of bacteria liquid was absorbed over a specific period of time and the absorption of OD_600_ is measured. There was no significant difference compared with the growth curve of the bacteria under dark conditions (Fig. 2c), indicating that the presence of blue light did not affect the normal growth of the strain. Relying on the same method, the *Lactococcus lactis* strain (LL-pDawn-GFP) stably expressing GFP was constructed and the growth curves of the two light-responsive verification strains under different powers under 473 nm blue light irradiation were measured (Fig. 2f, g). The results showed that the growth curve of the strain in the range of optical power 0~2.5 w did not show significant interference. MTT (3-(4,5-dimethylthiazol-2-yl)-2,5-diphenyltetrazolium bromide) assay for apoptosis further validates the biological safety of different power light sources (Fig. 2h). This provided great convenience for our subsequent plasmids construction. Obviously, precise spatiotemporal control is one of the key advantages of using light in the manipulation of biological systems due to the nature of light’s selective intervention at any time^24^. Photoresponsivity engineered strains are also expected to become a biologically and environmentally friendly probiotic.

### Delivery of Chitosan-Sodium alginate cross-linked hydrogel-loading engineered strains

**Fig. 3:**
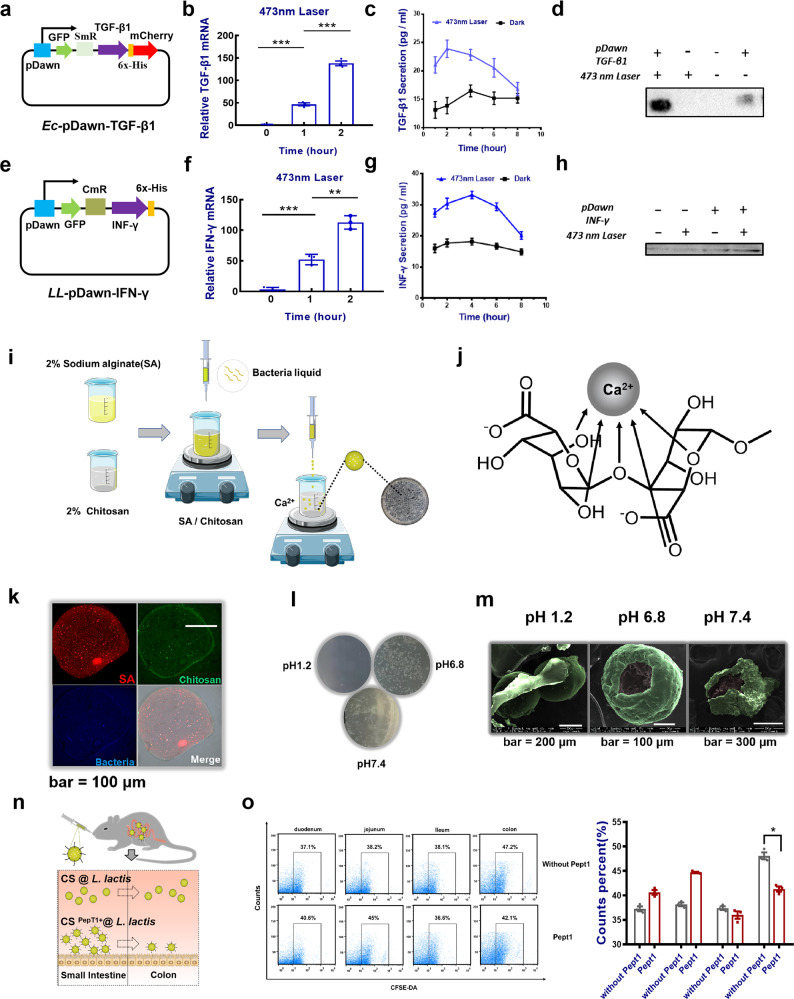
Delivery of Chitosan-Sodium alginate cross-linked hydrogel loading engineered strains. **a** Construction of pDawn-TGF-β1 engineered *E. coli*. **b** Real-time PCR analysis of the TGF-β1 mRNA expression levels induced by blue light (473 nm) (*n* = 3). **c** ELISA measurement of TGF-β1 levels secreted by engineered *E. coli*. **d** Protein levels of TGF-β1 detected by western blotting (the uncropped image of blot was shown in the Supplementary Fig. 18a). **e** Construction of pDawn-IFN-γ engineered *L. lactis*. **f** Real-time PCR analysis of the IFN-γ mRNA expression levels induced by blue light (473 nm) (*n* = 3). **g** ELISA analysis of IFN-γ levels secreted by engineered *L. lactis.* **h** Protein levels of IFN-γ detected by western blotting (the uncropped image of blot was shown in the Supplementary Fig. 18b). **i** The in vitro packaging process of engineered bacteria by hydrogel. **j** The mechanism of alginate-chitosan hydrogel. **k** The morphological characterization of hydrogel microspheres by confocal microscopy. **l** The hydrogel microspheres containing engineered bacteria were immersed in artificial simulated gastric fluid SGF (pH 1.2) and artificial simulated intestinal fluid SIF (pH 6.8 and 7.4) for 3 h, and then an appropriate amount of solution was selected for plate coating culture growing situation. **m** Characterization of microspheres appearance in different pH buffers by SEM. **n** Functional scheme of small intestine-targeting peptide Pept1. **o** Flow cytometry detection of colony flora in intestinal mucosal surface of different sections of mice for verification of the important role of targeting peptide (*n* = 5). Data are expressed as the mean ± SEM; *P < 0.05, **P < 0.01, ***P < 0.001.

Based on this, two common types of immune small molecules as target products, transforming growth factor-β1 (TGF-β1) and interferon-γ (IFN-γ), were applied to the pDawn blue light-responsive plasmid system to construct two engineered strains (Ec-pDawn-TGF-β1 and LL-pDawn-IFN-*γ*) (Supplementary Fig. 1 and Fig. 2). The plasmid structure were shown in Fig. 3a, e. The two engineered strains were shaken to the logarithmic growth phase under appropriate conditions and then induced by blue light for 2 h. Reverse-transcription PCR and western blotting results showed that both TGF-β1 and IFN-γ were highly expressed compared with dark control groups (Fig. 3b–d,  f–h). Similarly, enzyme-linked immunosorbent assay (ELISA) was also performed on the corresponding products of the bacterial solution with increased light induction time. As illustrated in Fig. 3c, g, the expression of the product generally increased after induction by light at the early stage. Over time, the expression of the product gradually decreased. The results suggested that the strain-induced effect could exist for a certain period of time and the engineered strain was not suitable for long-term continuous lighting. So far, the feasibility of the whole system has been preliminarily verified in vitro. Surely, engineered strains must undergo a series of conditions before they colonize and play a beneficial role in the intestine. These conditions, especially the low pH of the stomach, will cause a considerable loss of the viability of strains. To ensure that considerable engineered strains remain in the intestine, it need to choose a suitable method for strain encapsulation^25^. Alginate, a linear polysaccharide consisting of (1–4)-linked β-d-mannuronic acid (M) and (1–4)-linked α-l-guluronic acid (G) residues, has been widely studied as a microbial encapsulation capsule, owing to its ability to make mild gum conditions in contact with divalent cations (e.g., calcium chloride)^26,27^ (Fig. 3j). Previous studies have reported that alginate has superior strain encapsulation effects. It can protect the strains from corrosion in strong acid environments, whereas it can release a number of active strains in the milder intestine. The only regret is that the unstable nature of alginate limits its individual applications. Chitosan is a natural basic linear cationic polymerized polysaccharide with good biocompatibility. It can react with alginate to form a water-insoluble biofilm^28–30^. A stable gel material Chitosan-Sodium alginate (CS) was formed by mixing and stirring at room temperature, providing an ideal choice for in vivo delivery of engineered strains. It is also to address the issue that the colonization efficiency of oral delivery engineered strains in the intestine of mammals is limited by various physical and chemical and enzymatic factors in the digestive tract. The detailed preparation process is shown in Fig. 3i. Therefore, the following method is used to prepare CS composite hydrogel microspheres, which are easier to be taken orally to wrap the engineering strain. Confocal images showed the structure of hydrogel microspheres by marking different components (Fig. 3k). In addition, the artificial gastrointestinal buffer simulated in vitro was cultured by extracting the equivalent amount after the microsphere was soaked for a certain time. The plate culture results showed that the release of encapsulated strains from microspheres was extremely low under acidic conditions (Fig. 3l and Supplementary Fig. 6). Scanning electron microscopy images were used to characterize the morphology of hydrogel microspheres at different pH environments. The vitality of the released strain in an approximately neutral buffer environment was also confirmed (Fig. 3m). In short, the controlled release characteristics of microspheres met the experimental needs of oral delivery. That is, the near-neutral buffer environment (simulating small intestinal fluid and large intestinal fluid) is more suitable for CS microsphere-releasing strains. Considering that there are more specific requirements for the location of engineering strain colonization in different scenarios, e.g., the engineered strain LL-pDawn-IFN-γ needs to increase the proportion of colonization in the small intestine segment. To this end, modification of small intestine-targeting peptides Pept1 on its surface can further help the microspheres target to the small intestine, allowing more engineered strains accurately colonize in the small intestine (Fig. 3n, o). In addition, the entire material system is fully biosafe (Supplementary Fig. 3). To the end, the secretion of “modification of secretory photoresponse engineered bacteria” had been built.

### Characterization and application of UCR-doped microspheres engineered bacteria

**Fig. 4:**
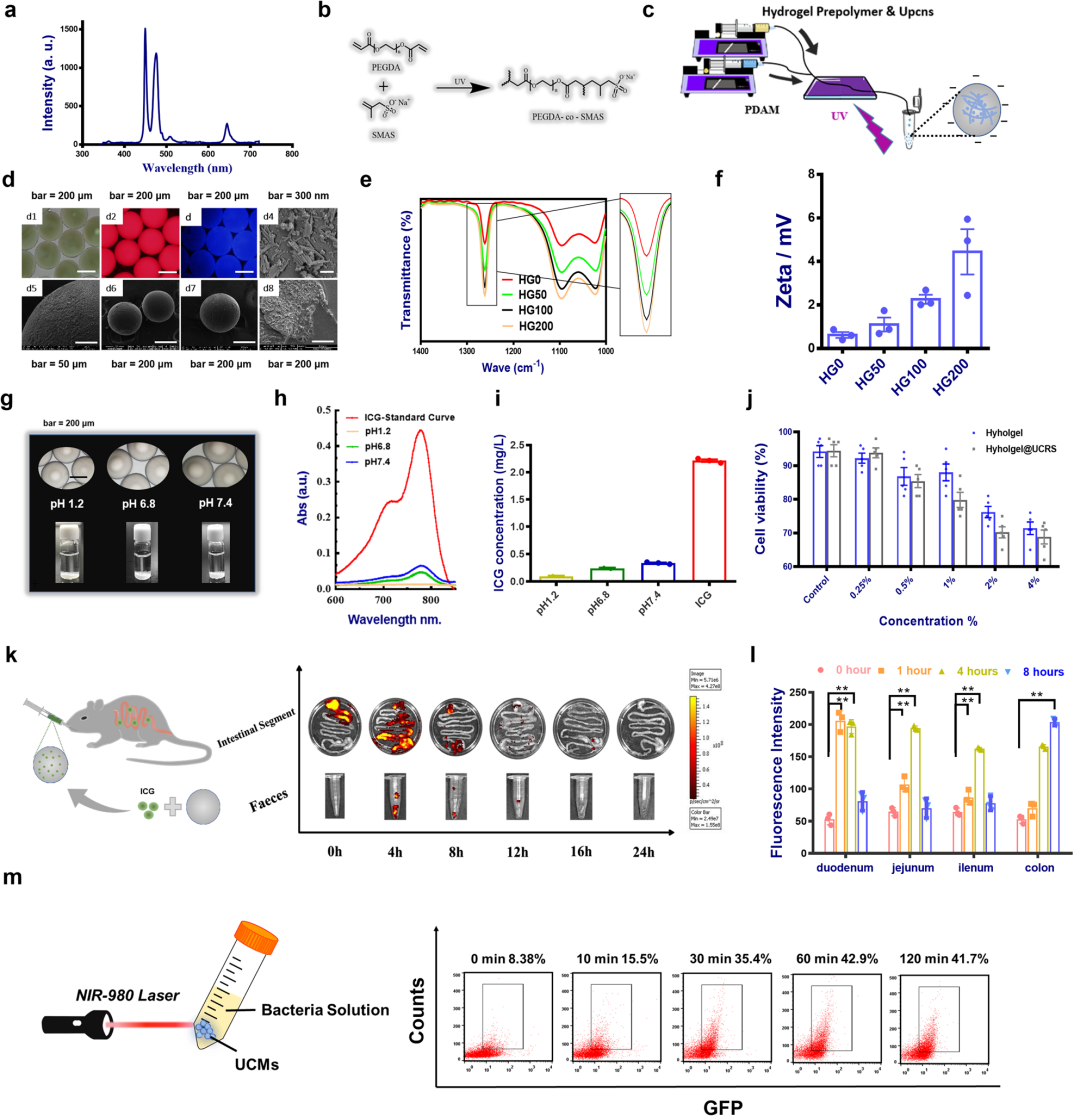
Modification of secretory light-responsive engineered bacteria. **a** The emission spectra of blue light emitted UCRs. **b** The principle of PEGDA-co-SMAS hydrogel cross-linking. **c** The scheme of PEGDA-co-SMAS hydrogel preparation. **d** The morphology of UCRs-doped microspheres. **e** Fourier-transform infrared spectroscopy (FITR) spectrum of PEGDA hydrogels grafted with SMAS of different contents. **f** The zeta potential of PEGDA hydrogels grafted with SMAS of different contents (*n* = 3). **g** The images of PEGDA-co-SMAS hydrogel microspheres containing ICG, which were immersed in artificial simulated gastric fluid SGF (pH 1.2) and artificial simulated intestinal fluid SIF (pH 6.8 and 7.4) for 3 h. **h** UV-visible spectrophotometric analysis of released ICG in different pH buffers. **i** The quantitative detection of released ICG. **j** Cell viability of different doping ratios of ICG and PEGDA hydrogel (*n* = 5). **k** Analysis of in vivo retention time of PEGDA-co-SMAS hydrogel microspheres containing ICG in different intestinal segments. **l** Mean fluorescence intensity of ICG in different intestinal segments. m)GFP expression (*n* = 5). Data are expressed as the mean ± SEM; *P < 0.05, **P < 0.01, ***P < 0.001.

As the sensitivity of blue light-sensing effect has been verified, the operation of the external light control system has become the next focus. Side effects on rhythm, sleep, and consciousness from visible light, especially blue light, have been reported in neuroscience in mice^31,32^. Therefore, it is necessary to find a more biologically safe alternative body light source. According to the existing synthesis methods, we have successfully synthesized UCRs with 980 nm excitation characteristics as key components for blue light generation^13^ (Supplementary Fig. 7). Its excellent photoluminescence properties can convert long-wavelength near-infrared light into locally efficient visible blue light (Fig. 4a). For the basic consideration of material safety, especially in the special microenvironment of the intestine, the UCRs were encapsulated in a polyethylene (glycol) diacrylate co-sodium methacrylate sulfonate (PEGDA-co-SMAS) microgel to form UCR-doped microspheres (UCMs). We developed a set of microspheres microfluidics equipment. The ultraviolet (UV)-curable hydrogel was prepared by using the compatibility difference between internal and external phase liquids (Fig. 4c). Such microspheres can retain the luminous properties of the UCRs, while preventing its toxic effect on the intestine itself due to release. PEGDA is a blank slate hydrogel that gels rapidly at room temperature in the presence of a photo-initiator and UV light^33^. They are hydrophilic, elastic, and can be used to load a variety of biological molecules. It is difficult to adsorb some adhesion proteins due to the strong hydrophilicity, limiting its application. Researchers have attempted to graft functional adhesion peptides on the surface of gels to improve material properties. In this study, we grafted a charged small molecule SMAS to the PEGDA backbone. By adjusting the amount of SMAS, the content of adhesion proteins on the gel surface can be promoted, thereby improving the biological properties of PEGDA gel^34^. In this study, the charged gel facilitated its adsorption on the intestinal mucosa. Its preparation and cross-linking principle were shown in Fig. 4b. Fourier-transform infrared spectroscopy experiments demonstrated the success of the special functional groups incorporated into small molecules (Fig. 4e). The subsequent swelling rate test, morphology and potential characterization of microspheres, and cytotoxicity test, respectively, illustrated the rationality and necessity of its application (Fig. 4f, j and Supplementary Fig. 5).

If the blue light generated by UCMs exposed to 980 nm laser, inducing gene expression in vitro is a focus. Two types of engineered strains (*E. coli* and *L. lactis*) carrying GFP tags were co-cultured with UCMs (2 mg/mL) under 980 nm laser (2 mW mm^−2^, 20 Hz). The flow cytometry results showed that the expression of GFP increased and the expression efficiency was similar to the direct blue light (Figs. 4m and 2d). These results were consistent with the direct blue light, confirming the feasibility of the UCMs with near infrared (NIR) light method and the potential of triggering downstream gene expression as a light source in vivo.

Based on the successful construction of the system above, in vivo implementing should be considered. Similar to the delivery of engineered strains, the oral delivery of UCMs also needs to consider the stability in different intestinal environment. Simulated gastric fluid (SGF) and simulated intestinal fluid (SIF) remain the standard for in vitro validation experiments. As shown in Fig. 4g, the morphology of material particles was reflected by different pH environments, demonstrating the unequal stability in different conditions. Next, visualization of in vivo delivery was convenient to explore the function of this system. A near-infrared fluorescent dye indocyanine green (ICG) was selected as a hydrogel-encapsulated object used in biomedical researches^35,36^. Compared with ICG standard products, the release amount was very low after soaking in the environment of SGF and SIF for a certain time (Fig. 4h, i and Supplementary Fig. 8). The cell cytotoxicity experiments indicated the safety of the delivery materials (Fig. 4j). After that, these dye-encapsulated microspheres were orally delivered to mice C57BL/6 at different time periods. In vivo images of dissected intestinal segments and stool samples showed that the above particles had good delivery effect and were basically emptied within about 12 h (Fig. 4k, l). These data showed that microspheres could be completely emptied from the intestines after a certain amount of time and did not affect normal physiological activity. To validate the safety of the system, the main organ and intestine segments of mice were collected for hematoxylin and eosin (H&E) staining. Results showed that compared with the untreated control group, the use of “UCMs + NIR” did not cause any organ damage. Intestinal epithelial cell (IEC) status was also unaffected. This further indicates that the system has good biocompatibility.

### NIR light-induced TGF-β1 secreting *E. coli* relieves UC in mice

**Fig. 5:**
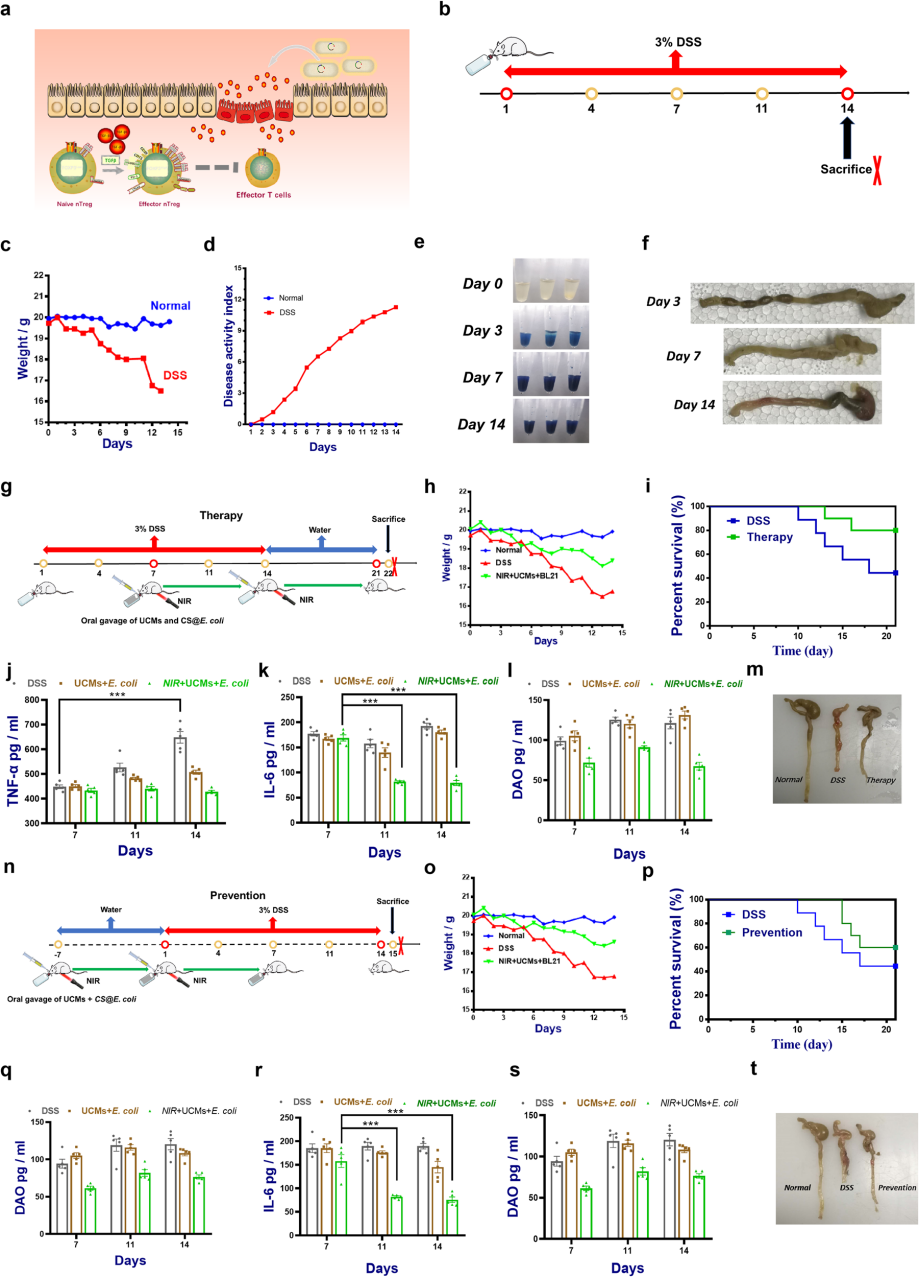
Efficacy of TGF-β1 secreted by light-responsive engineered *E. coli* on ulcerative colitis. **a** Mechanism of TGF-β1 inhibiting inflammation. **b** Schematic diagram of DSS-induced ulcerative colitis. **c** Weight changes within 14 days of drinking 3% DSS solution. **d** Changes in disease activity index (DAI). **e** Fecal occult blood test in mice. **f** Macroscopic appearance and colon length were measured in each group of mice at different points in time. **g** The scheme of ulcerative colitis treatment procedures. **h** Body weight changes. **i** The percent survival of mice with different treatments. The levels of representative inflammatory cytokines including (**j**) TNF-α, (**k**) IL-6, and (**l**) DAO in colonic tissues (*n* = 5). **m** The colon length was measured in each group of mice. **n** The scheme of ulcerative colitis prevention procedures. Changes in the (**o**) mouse body weight and (**p**) the percent survival of mice during 21 days of prevention. The levels of representative inflammatory cytokines including (**q**) TNF-α, (**r**) IL-6, and (**s**) DAO in colonic tissues (*n* = 5). **t** The colon length was measured in each group of mice. Pictures are the representative images from the results. Data are expressed as the mean ± SEM; *P < 0.05, **P < 0.01, ***P < 0.001.

We carried out further animal experiments to evaluate the efficiency of the above structured systems. The UC is a common intestinal immune inflammatory disease that can lead to chronic inflammation of the intestines for a long period. The characteristics of the small intestine in different sections were shown in the Supplementary Fig. 11, among which the colon was most affected by immune overshoot. Compared with the application of anti-inflammatory drugs, we hoped that the continuous secretion of endogenous immune molecules could be used to improve the adverse inflammatory microenvironment to achieve the goal of delaying the incidence of colitis. Previous researches have reported that extracellular vehicles with TGF-β1-dependent immunosuppressive activity were produced by IECs^36^ under physiological conditions^37,38^. At the same time, considering the previous research on the treatment of intestinal flora and inflammation, we hope to develop a new treatment plan around the microbiota. TGF-β1 would reduce the severity of UC by inducing regulatory T cells and immunosuppressive dendritic cells^39^ (Fig. 5a). The modified engineered *E. coli* induced growth under 980 nm light and dark conditions, including the addition of the same amount of hydrogel packets of rare-earth conversion particles. After 0–8 h of 37 °C thermostatic shake bacteria experiment, the TGF-β1 content was measured by ELISA. It has shown that higher secretion can be induced under near-infrared light conditions.

Dextran sulfate sodium salt (DSS) disrupts the mucosal barrier of the colon, causing intestinal inflammation, weight loss, and other clinical symptoms, thus becoming a common agent for clinically induced colitis^40^. C57/BL6 mice in the experimental group were orally treated with DSS (Fig. 5b) and a significant weight loss was observed from the day 4. Disease activity index (DAI) score is an indicator of the severity of colitis, based on weight loss, stool blood, and stool consistency. More weight loss, higher DAI scores, and shorter colon length were observed in DSS-treated mice in comparison with the normal group treated with water until day 14 (Fig. 5c, d, f). In the stool crypto-blood test, it could be found that the blood volume of mice treated with oral DSS reagent sat situ increased significantly (Fig. 5e). On the basis of the mature model construction, we began to study the practical application of the structured system in the treatment and prevention of diseases. As shown in the diagram Fig. 5g, n, the treatment intervention of the mouse with colitis was performed at different times. Three hours after oral administration of engineered strains capsules and rare-earth upconversion capsules, mice were exposed to 980 nm light. The overall change trend of the weight values of mice (*N* = 8) in different groups (Fig. 4h, o) and the percentage change in the mortality of mice caused by the onset of UC shown in the survival curve (Fig. 5i, p) observed “NIR + UCMs” treatment method helps to delay the progression of UC. To determine the anti-inflammatory effect, serum TNF-α and IL-6 expression levels were evaluated at different periods (Fig. 5j, k). The data indicated that mice with colitis treated above had lower inflammatory factor expression. Identically, in the case of pre-treatment, the occurrence of inflammation in healthy mice was continuously inhibited and delayed (Fig. 5q, r). As a basic feature of inflammation, intestinal permeability was evaluated by evaluating changes in plasma DAO content (Fig. 5l, s). The change in the content of DAO was similar to the index of inflammatory factors as expected in the experiment. At the same time, we tracked the statistics of the treatment situation. The colon color of the mice treated with colitis was more normal and the length atrophy was worse than that shown in the pictures (Fig. 5m, t). H&E staining of intestinal sections showed that DSS-induced colitis exhibited notably damages in colon structure with epithelial disruption, goblet cell depletion, and significant granulocyte infiltration (Supplementary Figs. 16 and 17). Immunohistochemistry analysis also showed the decreased expression of TGF-β1 and p-ERK. The exocystiation of intestinal IECs had a better repair effect on the intestinal mucosa in the coordination of TGF-β1 (Supplementary Figs. 16 and 17). Changes in p-ERK content also reflected the role of TGF-β1 downstream signaling pathways. Combined with the above data, the system achieved the initial delay and prevention effect in the treatment and intervention of UC in mice.

### NIR light-induced IFN-γ secretion by engineered *L. lactis* for anti-tumor immune effect in mice

**Fig. 6:**
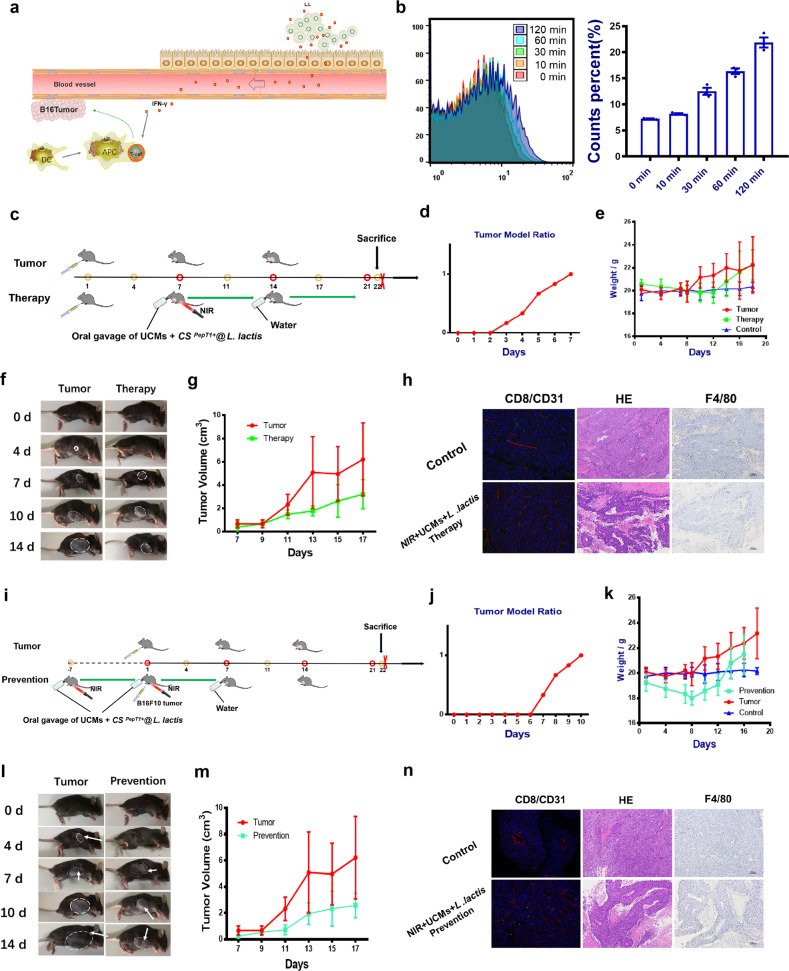
Inhibitory effect of IFN-γ secreted by light-responsive engineered lactic acid bacteria on distal subcutaneously transplanted malignant tumors. **a** Mechanism of IFN-γ inhibiting tumor progression. **b** Changes of IFN-γ expression over time under the induction of NIR 980 nm laser (flow cytometry monitoring of fluorescent labels) (*n* = 3). **c** The scheme of treatment procedure. **i** The scheme of prevention procedure. **d**, **j** The rate of B16F10 tumor formation rate over time. **e**, **k** Mice weight changes in different groups (*n* = 6). **f**, **l** The representative photos of tumor-bearing mice. **g**, **m** Tumor volume changes in different groups. **h**, **n** Tumor H&E staining of different groups. Immunofluorescence assay and IHC detection of macrophage marker F4/80 characterize tumor tissue maturity. Data are expressed as the mean ± SEM. *P < 0.05, **P < 0.01, ***P < 0.001.

Based on the inhibitory effect of intestinal inflammation, we hope to further explore the immune-assistive effect of this method. To this end, we constructed a model of B16F10 subcutaneous tumor to explore the far end of systemic immunity. Immune molecule IFN-γ is known to inhibit tumor cell growth and raise immune cells^41,42^ (Fig. 6a). We used this feature to select the more expressed *L. lactis* as the chassis modified into an engineered bacterium that could be specifically photo-controlled secretion of IFN-γ with 980 nm light induction to verify its in vitro secretion effect (Fig. 6b). Similar to previous treatments, we selected to orally enclose mice with engineered *L. lactis*-degradable hydrogel and microencapsulated rare-earth upconversion materials one week before and after subcutaneous injection of melanoma tumors. The specific animal experiment plan is shown in Fig. 6c, i, which are divided into “UCMs + NIR” intervention treatment after tumor onset and before tumor cell injection. In this experiment, the surface of the biodegradable hydrogel (CS alginate) was modified with a target peptide Pept1 to ensure that more highly active strains were effectively released in the small intestine segment. Take the day of tumor cell injection as the “start date” in both application scenarios. After 7 days, the tumor volume of the mice in the model group injected under the arm generally reached the observation requirements and the relevant indexes were measured immediately. By tracking and observing the degree of change in solid tumors and the living conditions, we found that the growth of tumors was significantly inhibited in mice with therapeutic intervention. This could be reflected in the course of changes in body weight, survival status, and tumor growth (Fig. 6d–g, i–m and Supplementary Fig. 12). Further evidence supported the observation of immune factor analysis of serum samples and observation of tumor tissue sections (Fig. 6h, n). Analysis of the results of histopathology and immunohistochemistry showed that the experimental group had a more pronounced effect on tumor cell apoptosis. From this we speculated that this immune effect caused by intestinal strains and affecting subcutaneous tumors had a certain systemic.

## Discussion

The above experiments fully demonstrate that the engineered probiotics could achieve oral colonization by oral delivery effectively, and utilize the symbiotic relationship between the host and microorganisms, thereby improving the immune environment of the intestine and the whole body with the help of specific strains secretions. The important role of intestinal microbiota has been receiving more and more attention in the medical and health field since the concept was introduced. Symbiotic bacteria can induce specific protein synthesis by directly adhering to the intestinal epithelial mucosa, thereby activating the intestinal lamina propria immune cells to play a role. Recent studies have shown that, in addition to the direct contact pathway of adhesion, commensal bacteria can indirectly regulate the status of innate immune cells through their own secretions, thus regulating the development and function of the immune system at the epigenetic level, such as short-chain fatty acids^5,43,44^. However, manipulating the expression of heterologous genes in artificially modified probiotics with spatiotemporal accuracy is still a huge challenge. As the active small molecules with immune effects in the body, the premise of their normal function is to ensure that the content is in a certain suitable range. If the expression level of the target gene cannot be effectively and precisely regulated, its role will be unknown. This also limits the clinical promotion and application of engineered strains to a certain extent. In this study, we constructed engineered *E. coli* strain Ec-pDawn-TGF-β1 and engineered *L. lactis* strain LL-pDawn-IFN-γ, respectively. The both strains can initiate gene expression in response to visible blue light in vitro. In vivo, NIR light (2 mW mm^2^ at 20 Hz), which has stronger tissue penetration ability, can successfully induce gene expression of engineered strains that colonize the intestine with the help of luminescence conversion of UCRs. Among them, UCRs are packaged in UCMs and also delivered orally through the gastrointestinal tract as an important optical conversion element. Therefore, the NIR light that avoids the negative effect of visible light on the nervous system and the UCMs that can be emptied from the body within 20 h together guarantee the safety and feasibility of the “UCMs + NIR” application approach. This strategy to control the expression of heterologous genes of engineered bacteria in vivo provides important ideas for effectively improving the immune status of the body.

In the specific work, we selected two typical immune-related disease models of UC and subcutaneously transplanted tumors as the main application scenarios of the established system. As a common intestinal immune hyperstimulation disease, antigen molecules in the intestinal cavity of patients with UC translocate to the lamina propria of the mucosa, and activate the lamina propria immune cells, producing a large number of inflammatory cytokines and inflammatory response mediators. They can cause intestinal mucosal barrier function damage by regulating tight junction protein expression^45,46^. The intestinal mucosa exposed to various antigens is continuously stimulated, forming a severe immune hyperstimulation response^47^. Although its pathogenesis is currently unclear, it is clear that reversing the state of an overwhelming immune response is an important strategy for delaying the course of UC. The engineered strain Ec-pDawn-TGF-β1 in this system can continuously secrete TGF-β1 with anti-inflammatory effect in response to light. Previous reports indicated that TGF-β1 was a new type of innate lymphocyte ILCregs that expanded and further exerted an important role of ILCregs in suppressing excessive natural immune response^48^. Therefore, controllable and engineered TGF-β1 can become a new solution to enteritis. Of course, compared with a single immune environment that affects the intestinal region, boosting systemic immune levels and adjuvant immunotherapy may be more worth looking forward. Malignant tumors are still incurable and life-threatening diseases. The recurrence of the tumor in situ and metastasis is still the main reasons for the clinical inability to cure the tumor. Although the emergence of immunotherapy has brought the possibility of a complete cure of the tumor in the future, it is limited to the tumor microenvironment. On the one hand, it provides a continuous source of nutrition and growth environment for tumors; on the other hand, it cooperates with tumors to escape immune resistance and assists tumor cells to metastasize and invade^49^. And IFN-γ can induce tumor blood vessel changes, leading to blood flow stagnation, thereby achieving the effect of inhibiting tumor growth^50^. It is well known that the intestinal region immune system is inextricably linked to the systemic immune system. If it can improve the vitality of the whole body’s immune system by utilizing the abundant blood flow and lymphatic drainage of the intestine with the help of delivery of immune protein molecules through the circulatory system, it will effectively assist tumor immunotherapy^51,52^.

Combined with the above results, it is clear that the upconversion optogenetic micro-nanosystem we constructed has the expected secretory regulation ability of engineered strains. Similarly, this concept can be further used by selecting a plasmid backbone with light-responsive start-up characteristics as an engineered bacteria transformation chassis, and add the corresponding target gene fragments as needed to achieve the purpose of establishing a “microbial pharmaceutical factory”. This concept is more safe than traditional oral and injectable administration methods, and optical control technology is easy to carry out for precious regulation. Nevertheless, our system still has some limitations that need to be addressed in future research. First, the experiment showed that the heterologous engineered microorganisms colonized in the intestines of mice could last for a month, but their light responsiveness to NIR declined with time. Second, there are certain differences in the degree of regulation of immune status among different mouse individuals, so whether to adjust the oral delivery amount of the engineered strain according to the physical condition of the mouse requires further follow-up investigation. In addition, the optical device equipment used in this study is not portable enough, and flexible light-emitting devices can be considered as an alternative in the future. In conclusion, this study provides a strategy to manipulate the expression of probiotic heterologous genes in the body to achieve the regulation of the immune status of the body and verify it. This may open a new chapter in the clinical application of microbial immunotherapy.

## Methods

### Reagents, light-responsive engineered strains and culture conditions

Molecular biology kits and chemical reagents were purchased from Thermo Scientific, Vazyme Biotech, Sigma-Aldrich, or Solarbio. The oligonucleotide primers involved in this study were synthesized by GENEWIZ, Inc. (Suzhou, China) (Table 1). The two main genetic engineered strains involved are *E. coli* BL21 and *L. lactis* NZ9000. Among them, the strain *L. lactis* NZ9000 is derived from strain *L. lactis* subsp. cremoris MG5267 but has a lacF deletion in the lactose operon. Both strains were maintained in Luria-Bertani medium supplemented with 50 μg/mL spectinomycin and M17 medium with 20 μg/mL chloramphenicol. All plasmids are constructed based on the blue light-responsive promoter pDawn. The blue light promoter pDawn was a gift from Ingmar Riedel-Kruse (Addgene plasmid #107742). The remaining functional element sequences including GFP, mCherry, ampicillin, and chloramphenicol resistance genes were gifts from Tao Sun (Tianjin University). The preferred codon for *E. coli* (TGF-β1) and the preferred codon for *L. lactis* (IFN-γ) were synthesized by Sangon Biotech. After completing the PCR amplification of the above-mentioned foreign fragments, phosphorylation modification of the 5′-end is performed by PNK enzyme (Thermo) and it is ligated to the corresponding plasmid backbone by T4 ligase (Thermo). Among them, *E. coli* DH5α strain was used as a host for the construction and amplification of recombinant *E. coli* BL21 plasmids. The recombinant plasmids of *L. lactis* MG5267 needs to be first transformed into TG1 competent cells with 2.5 KV, 1 pulse electric ratio, and then transformed into *L. lactis*. The strains were both kept at 37 °C and 30 °C with shaking culture (175 r/min) in the dark conditions before receiving the light induction.

**Table 1:** Main primers design.

Primers	Sequence (5′–3′)
LK-Gfp-R	AGACCCCCCCCCCCCAGACCCTTTGTATAGTTCATCCATGCCGAGTGT
BamHI-Gfp-F	CG**GGATCC**TCACACAGGAAACCTACTAGAATGGTGAGTAAAGGAGAAGAACTTTTC
XhoI-Mcherry-R	GTGGTGGTGGTGGTGGTGCTCGAGCTTGTACAGCTCGTCCATGCC
LKN-Mcherry-F	GGGTCTGGAGGCGGTGGTTCTGTGAGCAAGGGCGAGGAGGAT
LKN-Tgfb1-R	AGAACCACCGCCTCCAGACCCTGAACATTTACATGAACGAACAATCATATTTG
XhoI-RBS-SG-F	CTCGAGTTTGTTTAACTTTAAGAAGGAGATATACCATGAAAAAGACAGCTATCGCGATT
6xHis-F	ACTCGAGCACCACCACCACCA
IFN-xht-F	ATCAGGTGTTTATGCAATGAATGCAACAC
xht-GJ-F	GTTATTTTATCAGCTGCTGCTCCATTATCAGGTGTTTATGCATAAACAGCTGCTGGGATTACACTCG

### 473 nm and 980 nm light sources fabrication

A 473 nm blue light laser or a 980 nm NIR light laser (Changchun New Industries Optoelectronics Technology Co., Ltd) was connected with a signal generator (Tektronix) to achieve user defined-pulsed illumination. The frequency used for each light source in this paper was 20 Hz. The optical power was detected by an optical power meter (Daheng Optics) and the optical density of NIR light was kept at 2 mW mm^2^.

### Synthesis and characterization of UCRs

The UCRs have already synthesized as previously described^14,53^. Simply, NaOH (8 M) was mixed with EtOH and equal oleic acid under continuous agitation for 10 min at room temperature. Then, rare-earth solution (20 mM) and NH4F (2 M) were subsequently added and stirred for 30 min at 1200 r.p.m. to form a homogeneous solution. The mixture was then transferred to a reaction kettle and then heated at 180 °C for 12 h. The solution was allowed to cool overnight to room temperature. The UCRs in the precipitation were washed with EtOH and sterilized distilled water for five times, respectively. The UCRs were then kept in sterilized distilled water at room temperature for future use. The synthesized UCRs were characterized by transmission electron microscopy (TEMG2F20, Tecnai). Scanning electron microscope (SEM) observation was carried out at an operating voltage of 15 kV with a Nanosem 430 in CN mode. A fluorescence spectrophotometer (AvaSpecULS2048-USB2, Avantes) was employed to measure the fluorescence spectrum of UCNs using a 980 nm laser.

### Preparation and characterization of UCMs

The UCMs particles were designed and synthesized using a self-made orthogonal microfluidic system driven by two microsyringe pumps (Fig. 3c). In general, the ratio of the components in the designed PEGDA-co-SMAS hydrogel precursor prepolymer solution is PEGDA-700 : D2959 (2.5% w/v in HEPES Buffer) : SMAS (2 mM) : HEPES Buffer (20 mM) = 20 : 4 : 5 : 1. Among them, the preparation of D2959 should be fully stirred at room temperature in advance overnight. The UCRs solution were doped in the prepolymer to reach the final concentration of 10 mg/mL. During the preparation of the microspheres, the above solution mixture will flow out as an internal phase liquid from a 1 mL sterile syringe (protected from light) at a rate of 40 μL/min. The external phase liquid was simethicone flowing out at a rate of 1200 μL/min. Using the difference in polarity of the external and internal phase liquids, uniform droplets can be formed at the junction of the above two-phase liquids. During passing through the pipeline, an ultraviolet light (JINGKE) was used to excite the cross-link of the photosensitizer D2959 to form uniform UCMs. The particles were then collected and were rinsed for 5 times with sterilized distilled water. The UCMs were then kept in sterilized distilled water at room temperature for future use. To detect the hydrogel by SEM (XL-30, Philips Corp.), 1 mg hydrogel sample were dehydrated using freezing vacuum dryer (Song Yuan Freeze Dryer). SEM observation was then carried out at an operating voltage of 15 kV with a Nanosem 430 in CN mode, as previously described. A fluorescence inversion microscope system (Olympus, Japan) with 980 nm excitation mode was employed to detect the luminescence property of UCMs.

### Characterization of PEGDA-co-SMAS hydrogel materials

*Fourier-transform infrared spectroscopy*. After lyophilization for 24 h, the chemical structures of the hydrogels were characterized using a Fourier-transform infrared (2000, PerkinElmer, USA) spectrophotometer (*n* = 3).

*Zeta potential*. The hydrogel samples in each group (*n* = 3) were immersed in deionized water to achieve the equilibrium state and ground into fine particles. After drying in a vacuum oven, 10 mg of particles were weighed and dissolved in 1 ml of deionized water and the Zeta potential was measured by using dynamic light scattering on a Zetasizer Analyzer (Malvern).

*Swelling ratio*. To obtain the swelling balance of the hydrogel, the cut samples (*n* = 6) with a size of 1 cm × 1 cm × 1 mm were immersed in deionized water or phosphate-buffered saline (PBS) at 37 °C soak for 24 h, respectively. Then, excess water was removed with filter paper, and the swollen hydrogel (Ws, swollen weight) was weighed. The treated samples were placed in a 55 °C vacuum oven and dried for 24 h to achieve complete dehydration and weigh the dry weight of the sample (Wd, final dry weight). The calculation formula of the swelling rate is as follows: Swelling rate = (Ws − Wd)/Wd.

*Synthesis of CS* ^*PepT1+*^*@ E. coli (L. lactis*). Sodium alginate solution (2% in sterilized distilled water) was stirred continuously at 800 r.p.m. for 1 h at room temperature. During mixing process, *E. coli* and *L. lactis* were added drop by drop to the sodium alginate solution. The content of the *E. coli* and *L. lactis* inside the hydrogel conformed to Poisson distribution, so the concentration of the bacterial were kept to almost 5 × 10^9^ CFU/mL in sodium alginate solution. Use a sterile syringe to squeeze the above mixed solution dropwise into the 2% CaCl_2_ solution, and keep the distance between the needle tip and the surface of the calcium chloride solution at about 50 cm. After standing and hardening for 30 min, a large amount of uniform sodium alginate hydrogel microspheres can be obtained. Next, the chitosan solution (5 wt% in sterilized distilled water) mixed with PepT1 antibody (1 : 1000 v/v) were stirred at 4 °C for 1 h at 800 r.p.m. Next, the chitosan solution (5 wt% in sterilized distilled water) mixed with PepT1 antibody (1 : 1000 v/v) were stirred at 4 °C for 1 h at 800 r.p.m. The alginate microcapsules were then incubated with this chitosan solution at 4 °C for 1 h at 800 r.p.m. to form a chitosan and PepT1 shell. The gel was washed with sterilized distilled water at 1000 r.p.m./min for three times to remove extra polymer. The chitosan and sodium alginate hydrogel (CS ^PepT1+^@ *E. coli*/*L. lactis*) were then kept at acetic acid solution at 4 °C. Before use it was washed with sterilized distilled water at 1000 r.p.m./min for three times to remove extra acetic acid.

*Flow cytometry*. Flow cytometry measurements were performed using FACS Calibur instrument (BD Biosciences), with a minimum of 40000 cells and the data were analyzed using FlowJo V10. In vitro, the bacterial was collected at 8000 r.p.m. for 5 min, and washed with PBS for twice. In vivo, mice were killed and various intestine segments were taken out. The intestinal mucosa was collected and resuspended in PBS at the volume ration of 1 : 1. Precipitate was removed by centrifugation of 2000 r.p.m. for 15 min and bacteria was collected by centrifugation of 8000 r.p.m. at for 5 min. Then the sediment was fixed with 4% paraformaldehyde for 20 min and followed by PBS wash. The sediment was then resuspended in 1 mL PBS before flow cytometry analysis. All procedures were kept in low temperature.

*Animals*. We used wild-type female C57BL/6N mice (SPF (Beijing) Biotechnology Co., Ltd) in this study. All mice were weighted between 20 and 25 g (6~8 weeks years old) and were maintained on a 12 h light–dark cycle. All animal experiments were performed by the statutory requirements of People’s Republic of China (GB14925-2010). For engineered strains transplantation, mice were orally gavaged 200 μL of CS hydrogel in PBS that contained ~2 × 10^9^ CFU *E. coli* and *L. lactis*. This procedure was carried out at about 10:00 a.m. for two continuous weeks. Hair removal was performed on the abdomen of mice 4 h after oral administration, and irradiation with a pulsed NIR light source was continued for 1 h. Feces of mice were collected and autoclaved at 121 °C for 30 min to avoid environmental contamination.

*Mouse imaging in vivo*. We used healthy male C57BL/6 mice (Beijing Vital River Laboratory Animal Technology Co., Ltd) of 20–25 g body weight. The fluorescence imaging of the mice was performed with a small animal imaging system under 645 nm excitation. After administration of microgel@ ICG, taken the mice, organs (heart, liver, spleen, lung, kidney, and gut) and feces were imaged every hour.

*The mice model of UC and treatment*. The mice were given free access to 3% DSS (60316ES25) aqueous solution for 7 days. Using urine fecal occult blood test kit (60403ES60) injury of colon mucosa. Using upconversion optogenetic micro-nanosystem for 14 days of treatment. The recovery of colitis mice was observed by H&E staining of colon in mice. ELISA kits (Shanghai Huyu Biotechnology Co., Ltd) were used to detect levels in serum and intestinal tissue. Immunohistochemical data can further show the content levels of corresponding molecules and downstream factor indicators.

*The mice model of and subcutaneously transplanted tumors B16F10 and treatment*. For the tumor inoculation, each mouse was subcutaneously injected about 1 × 10^6^ B16F10 cells into the left flank region of the mice. Among them, the tumor cells of the prevention group were inoculated 1 week later than the treatment group. On the contrary, the oral administration of UCMs and strains of the group was 1 week earlier (Fig. 5c, j). At the same time, oral feeding and pulsed NIR light source irradiation treatment were carried out. Regularly acquiring data on mouse weight and tumor volume to judge the trend of tumor growth. Meanwhile, the level of macrophages in tumor was tested by immunofluorescence histochemistry staining (labeled by F4/80). Immunofluorescence and immunohistochemistry data analysis can also reflect changes in tumor spreading ability.

*Immunostaining*. For immunostaining of gut tissue, ~1 cm of appointed intestinal segment was taken out and the connective tissue of the ectotheca was carefully removed, the intestinal content was washed with sterile PBS for three times. All procedures were kept on ice. The tissue was then embedded in OCT (SAKURA) to make frozen section with thickness of 35 μm. Slices were incubated with corresponding antibody (1 : 200 dilution in tris-buffered saline (TBS)) at 4 °C overnight, followed by TRITC-conjugated goat anti-rabbit (1 : 500 dilution in TBS) at room temperature for 2 h. Finally, the mounting medium with 4′,6-diamidino-2-phenylindole was dropped onto the sample to prevent fluorescence quenching. Images were taken by A1 HD25 confocal microscopy (Nikon).

*GFP protein translation levels and His-tag measured by western blotting*. Western blotting analysis was done to detect the GFP or His-tag. After high-speed centrifugation, extracellular proteins were prepared and subjected to n-electrophoresis on a 12% gradient gel and transferred to a polyvinylidene difluoride (PVDF) membrane by semi-dry transfer method (22 V, 22 min). The PVDF membrane was blocked in 5% bovine serum albumin (BSA) for 1 h and then washed by TBST three or four times (5 min each time). The membrane was incubated in primary antibody (Anti-GFP or Anti-His-tag at 1 : 1000 dilution in 5% BSA) on a platform shaker at 4 °C overnight. The membrane was then washed three or four times by TBST again, and incubated with a secondary antibody (Sungene Biotech) in a ratio of 1 : 2000 in 5% BSA, and incubated at room temperature for 1 h. At last, the membranes were exposed to chemiluminescent substrate (Univ) imaged with an imaging station (biorad ChemiDoc MP) after the third washing by TBST.

### Schematic construction statement

The layout and design of all schematic diagrams involved in this manuscript were done by co-authors. Some medical image elements in the schematic diagram, such as cells, tissues, organs, etc., are provided by the Servier Medical Art (https://smart.servier.com) under CC BY 3.0 license (Figs. 1,  2d,  3i,  4c, m,    5a, and  6a, etc). Some of the image elements of chemical experiment instruments are produced by the PowerPoint plug-in ScienceSlide2016. Other graphic elements such as laser circuit diagrams, experimental mouse model diagrams, etc. were designed or drawn by the co-authors in this study.

### Statistics and reproducibility

All results presented in graphs show the mean data ± SEM. Two-tailed Student’s *t*-test was applied to analyze significant differences between groups (***P < 0.0001, **P < 0.001, *P < 0.05). *P < 0.05 was considered significant. No statistical method was used to predetermine sample size.

### Reporting summary

Further information on research design is available in the Nature Research Reporting Summary linked to this article.

## Supplementary information

Supplementary Information

Reporting Summary

## Data Availability

The data that support the findings of this study are available within the manuscript files or from the corresponding author upon reasonable request.
