# Author Correction: Upconversion optogenetic micro-nanosystem optically controls the secretion of light-responsive bacteria for systemic immunity regulation

**DOI:** 10.1038/s42003-020-01415-0

**Published:** 2020-11-05

**Authors:** Chun Yang, Meihui Cui, Yingying Zhang, Huizhuo Pan, Jing Liu, Shixing Wang, Ning Ma, Jin Chang, Tao Sun, Hanjie Wang

**Affiliations:** 1 School of Life Sciences, Tianjin University, 300072 Tianjin, China; 2 Tianjin Engineered Center of Micro-Nano Biomaterials and Detection-Treatment Technology, Tianjin Key Laboratory of Function and Application of Biological Macromolecular Structures, 300072 Tianjin, China; 3 Academy of Medical engineered and Translational Medicine, Tianjin University, 300072 Tianjin, China; 4 Center for Biosafety Research and Strategy, Tianjin University, 300072 Tianjin, China; 5 Laboratory of Synthetic Microbiology, School of Chemical engineered and Technology, Tianjin University, 300072 Tianjin, China

**Keywords:** Applied microbiology, Ulcerative colitis

Correction to: *Communications Biology* 10.1038/s42003-020-01287-4, published online 9 October 2020.

In the original version of the published Article, Fig. 2 was mistakenly replaced by another copy of Fig. 3.

This has now been corrected in the PDF and HTML versions of the Article.

