# Supplementary material for: Upconversion optogenetic micro-nanosystem optically controls the secretion of light-responsive bacteria for systemic immunity regulation
Source: Commun Biol. 2020 Oct 9;3:561. doi: 10.1038/s42003-020-01287-4 (PMC7547716; doi:10.1038/s42003-020-01287-4)
Supplement: Supplementary file 1 — Supplementary Information [file 42003_2020_1287_MOESM1_ESM.docx]

Supplementary material for

**Upconversion Optogenetic Micro-nanosystem Optically Controls the Secretion of Light-responsive Bacteria for Systemic Immunity Regulation**

Chun Yang,^1,2,^ ^#^ Meihui Cui,^1,2, #^ Yingying Zhang,^1,2, #^ Huizhuo Pan,^1,2^ Jing Liu,^1^ Shixing Wang,^5^ Ning Ma,^1,2^ Jin Chang,^1,2^ Tao Sun,^3,4, *^ and Hanjie Wang^1,2, *^

**^1^** School of Life Sciences, Tianjin University, Tianjin, 300072, China.

**^2^**Tianjin Engineering Center of Micro-Nano Biomaterials and Detection-Treatment Technology, Tianjin Key Laboratory of Function and Application of Biological Macromolecular Structures, Tianjin, 300072, China.

**^3^**Center for Biosafety Research and Strategy, Tianjin University, Tianjin, 300072, China.

**^4^**Laboratory of Synthetic Microbiology, School of Chemical Engineering & Technology, Tianjin University, Tianjin, 300072, China.

**^5^**Academy of Medical Engineering and Translational Medicine, Tianjin University, Tianjin, 300072. P.R. China

* Corresponding author. E-mail: tsun@tju.edu.cn and wanghj@tju.edu.cn

**Supplementary Table 1.** The full details of pulse 980nm NIR excitation light in animal experiment

| **Parameter** | **Value** |
| --- | --- |
| Frequency | 20 Hz |
| Light Intensity | 2 mW/mm^2^ |
| Duty Cycle | 50% |
| Pulse Width | 25 μs |


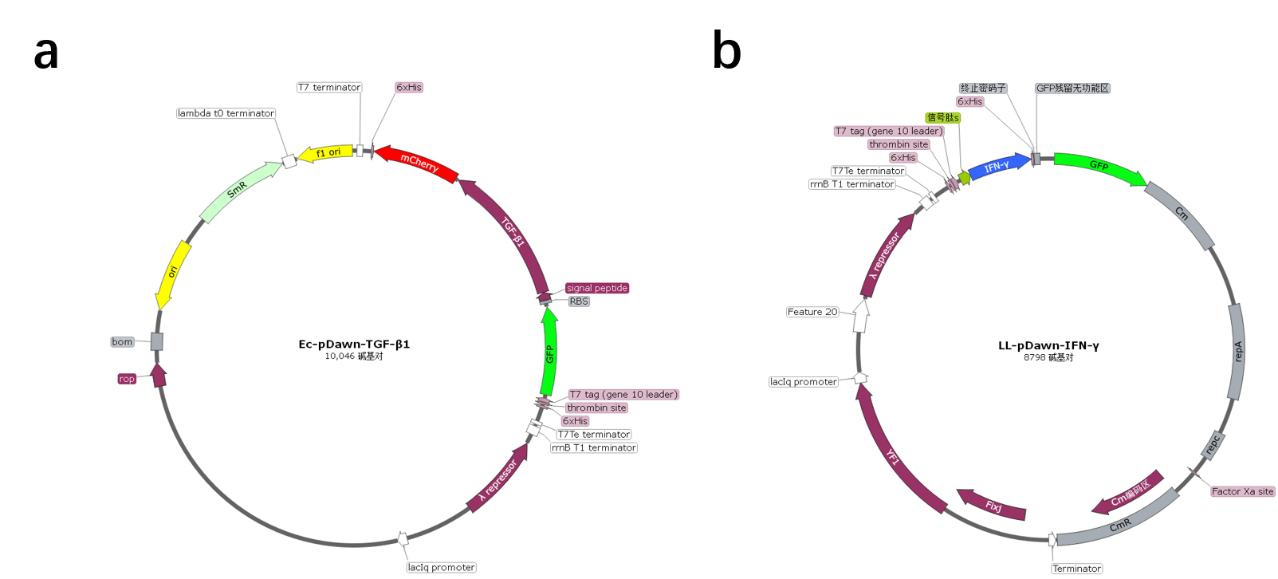


**Supplementary Figure 1.** (a) *E. coli* recombinant expression vector Ec-pDawn-TGF-β1 plasmid map. (b) *L. lactis* recombinant expression vector LL-pDawn-IFN-γplasmid map.


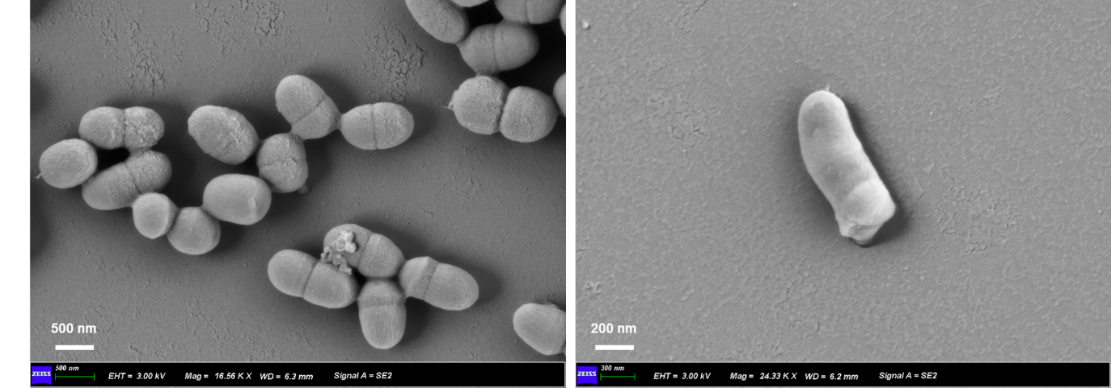


**Supplementary Figure 2.** Scanning electron microscope images of *L. lactis* MG5267 and *E. coli* BL21.The isolated and purified bacteria were picked into a shake flask containing 10 mL of LB liquid medium and cultured with shaking for 12 h. After culturing, 800 μL of the bacterial solution was sucked and centrifuged for 3 minutes (speed 3000 rpm). Take the centrifuged pellet and add 500 μL of 1×PBS to wash it 2 to 3 times. Subsequently, the bacterial solution was fixed with 4% glutaraldehyde for 2 hours and gradient centrifugation was performed with ethanol. Finally, add 200 μL of 100% tert-butanol at 4°C for 30 min, then dissolve in an appropriate amount of 100% tert-butanol and drop it on tin foil paper to deliver the sample, and then it can be detected by scanning electron microscopy.

.
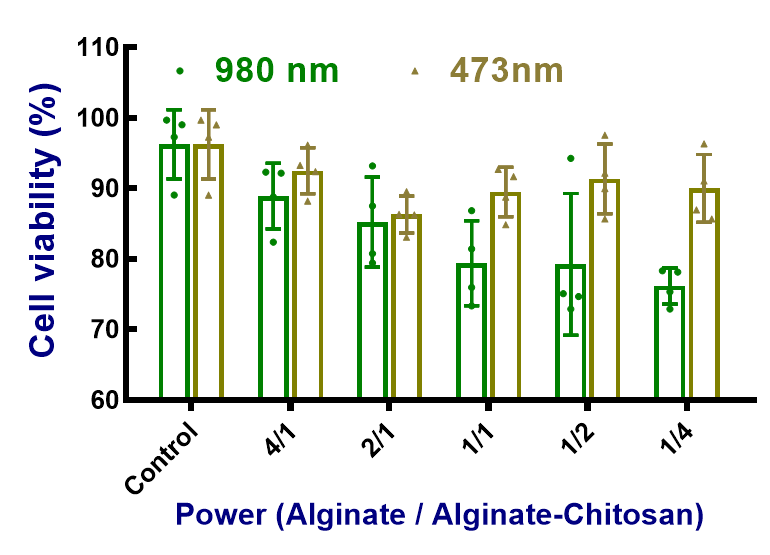


**Supplementary Figure S3.**  The results of cell experiments prove that whether alginate is cross-linked with chitosan will not increase cytotoxicity. The selected hydrogel material has good biological safety. The results of cell experiments prove that whether alginate is cross-linked with chitosan will not increase cytotoxicity. The selected hydrogel material has good biological safety. The data represent the mean and standard deviations of six biological replicates.(n=5)


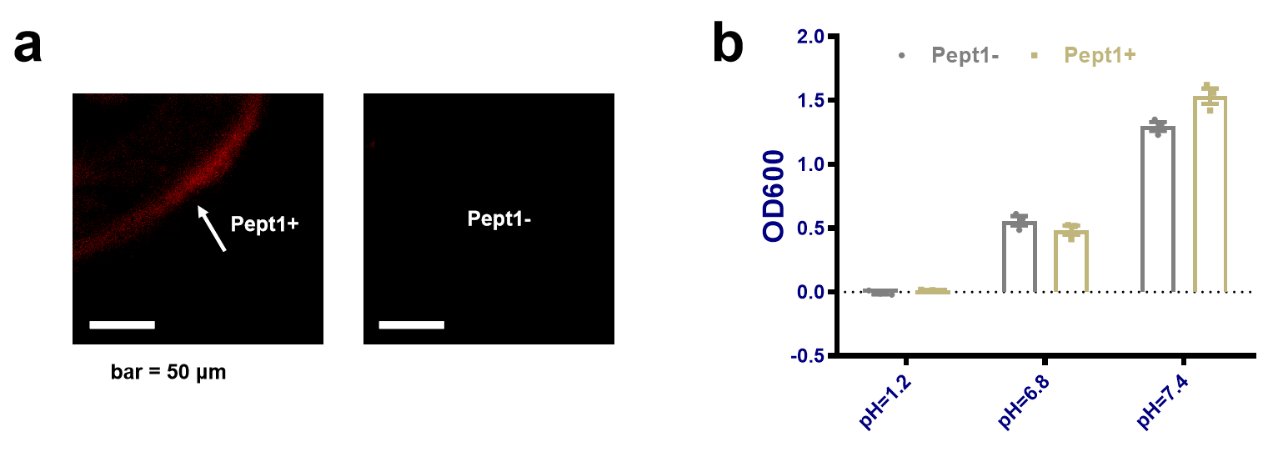


**Supplementary Figure 4.** (a) Confocal schematic diagram of small intestine targeting peptide Pept1. Immunofluorescence of PepT1, which was labelled with a rabbit anti-SLC15A1 polyclonal antibody (pAb) (red), in the chitosan layer of the CS hydrogel. White arrows indicate labelled PepT1 in the chitosan layer outside the CS hydrogel. (b) Release of simulated strains in vitro at different pH. The data represent the mean and standard deviations of three biological replicates. The simulated strains were packed into the CS hydrogel and immersed in chitosan buffer or buffers with various pH values for 1 h. Bacterial release was determined by measuring the OD600 of the supernatant.(n=5)


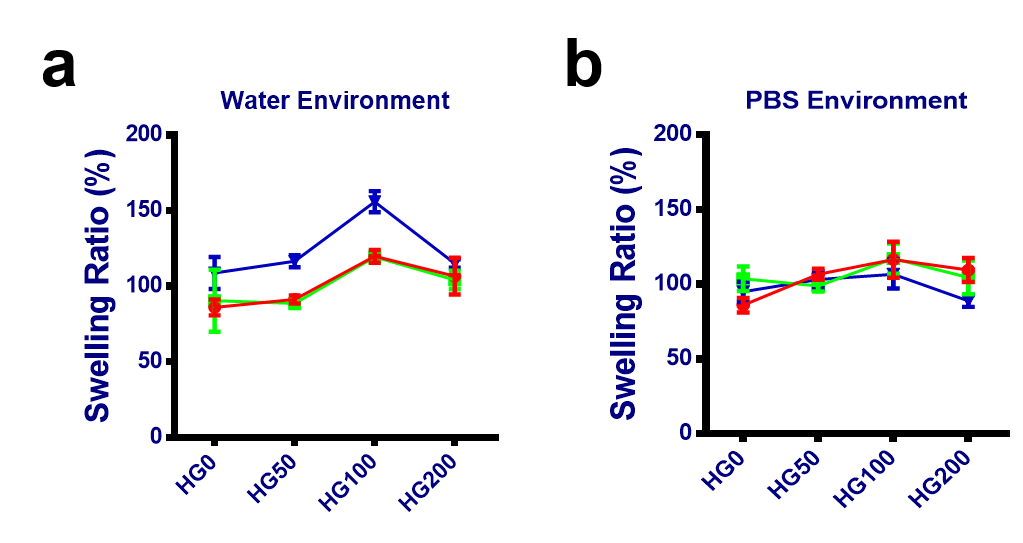


**Supplementary Figure 5.** Swelling performance test of PEGDA-c0-SMAS hydrogel microspheres (that is, UCMs loaded with up-converter rods) in aqueous solution and PBS buffer for 24 hours. Compared with aqueous solutions, the swelling of PEGDA-co-SMAS hydrogel microspheres with different potentials in PBS buffer is more stable. The data represent the mean and standard deviations of six biological replicates.


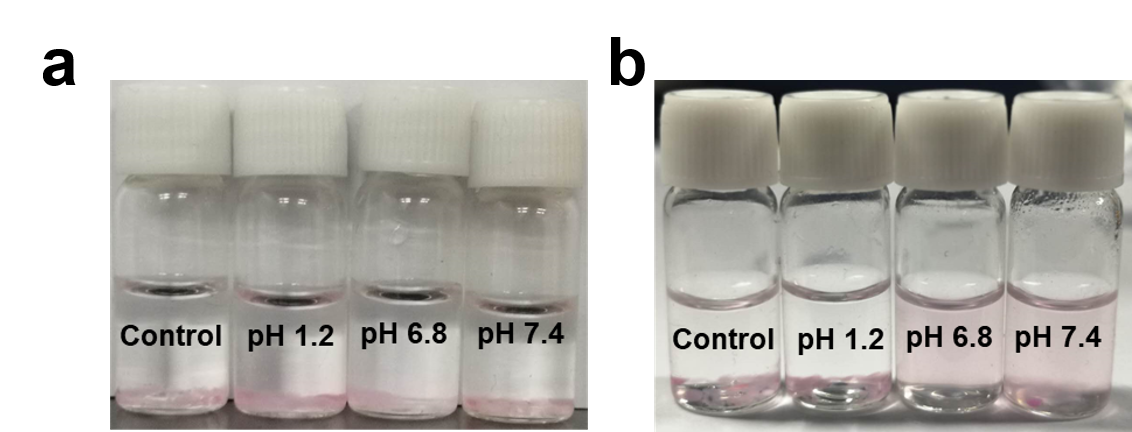


**Supplementary Figure 6.** Release of Simulated Strains in Vitro at Different pH——Characterization of Particle Integrity with Rhodamine Dye.

(a) Initial state of alginate-chitosan hydrogel microspheres in different pH buffers. (b) State of alginate-chitosan hydrogel microspheres after 4 hours in different pH buffers. The color change of different pH buffers can characterize the structural integrity of hydrogel microspheres CS. The deepening of the red in the solution indicates that the damage of the microspheres has increased.


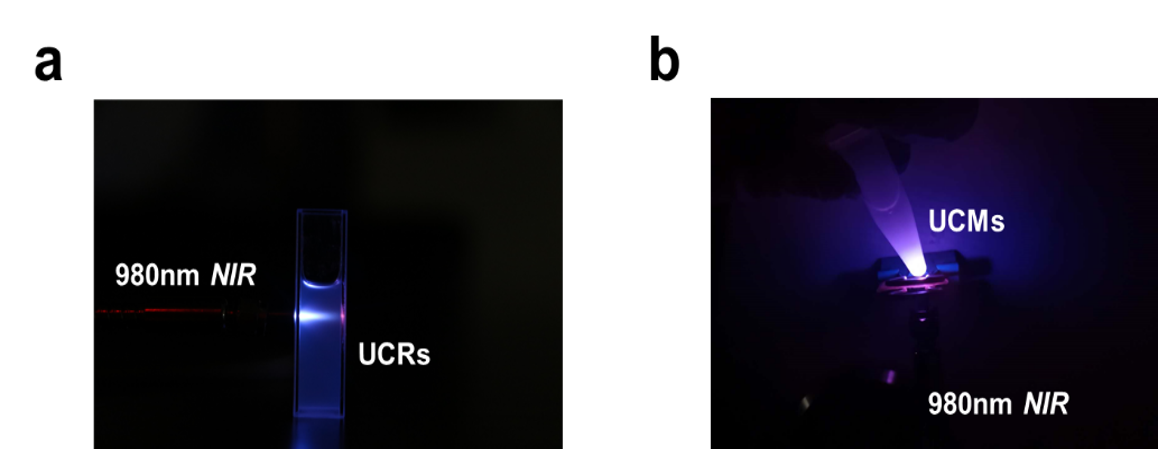


**Supplementary Figure 7.** The verification of UCRs and UCMs upconversion luminescence characteristics. UCMs, that is, PEGDA-co-SMAS loaded UCRs hydrogel microspheres, when 980 nm NIR light source is irradiated, the luminescence converted blue light brightness is not weaker than the brightness of UCRs solution at the same concentration.


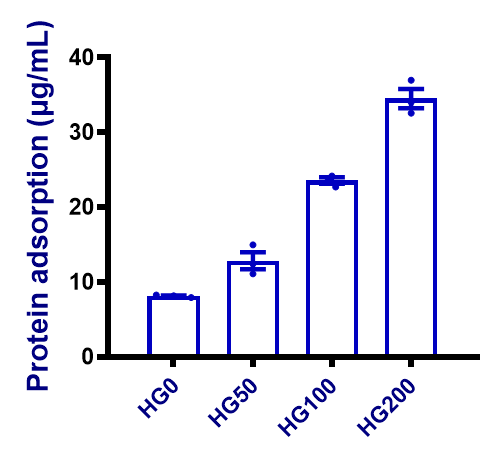


**Supplementary Figure 8.** Determination of protein adsorption rate of SMAS modified hydrogel PEGDA. The results show that as the SMAS concentration increases, the amount of protein adsorbed on the PEGDA surface of the charging gel increases. This means that the incorporation of negatively charged small molecule SMAS is beneficial to improve the biosorption capacity of the gel surface. The data represent the mean and standard deviations of six biological replicates.（n=3）


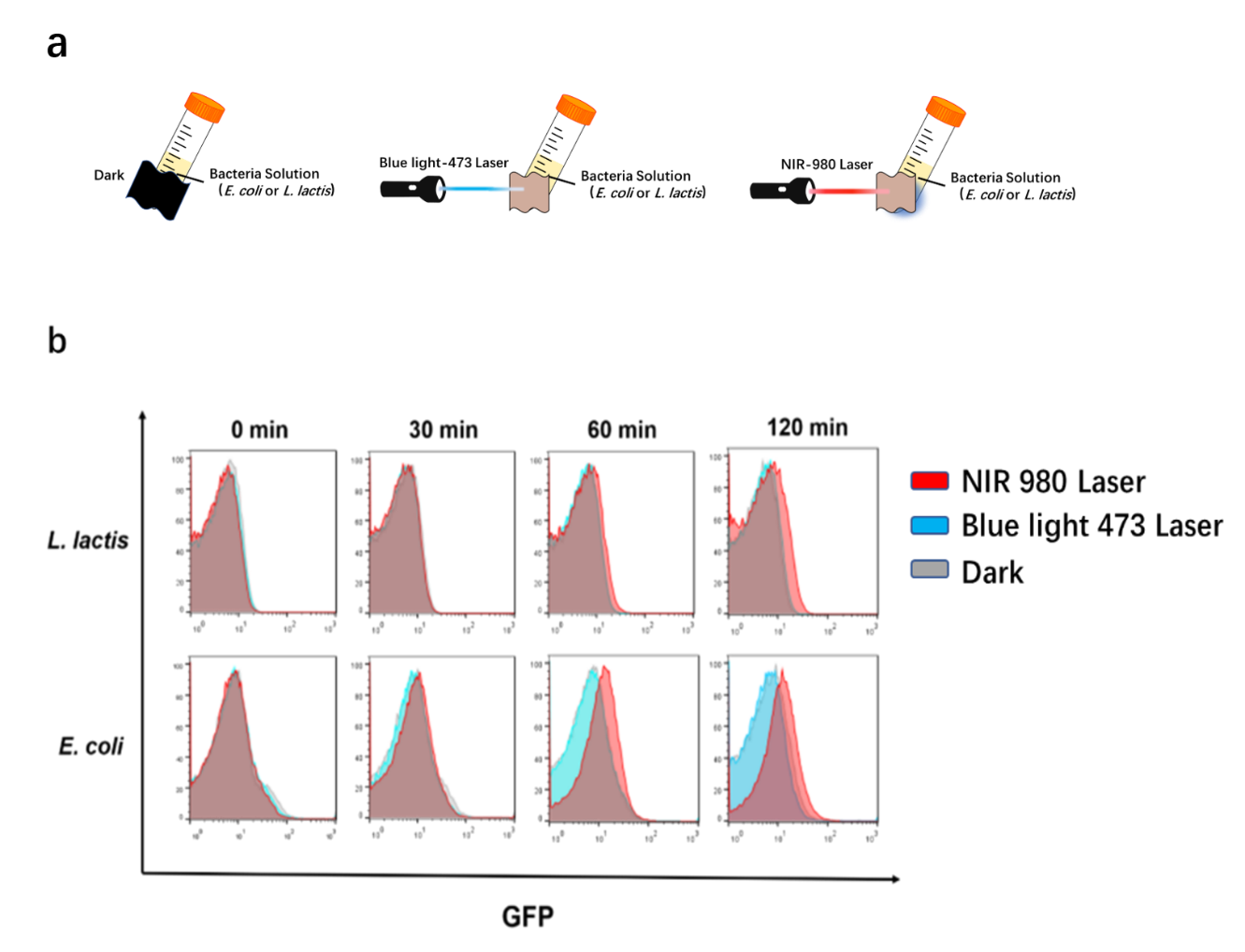


**Supplementary Figure 9.** Transdermal photoinduction simulation experiment of engineered Bacteria in vitro. (a) Schematic diagram of in vitro light-induced simulated tissue penetration experiment. (b) Both *E. coli* and *L. lactis* strains produced more GFP expression under the induction of NIR light source over time. On the contrary, the GFP content of the blue-irradiated group was not obvious compared with the dark and dark group increase. According to the above experimental results, it can be proved that NIR has a stronger tissue penetration effect than blue light.


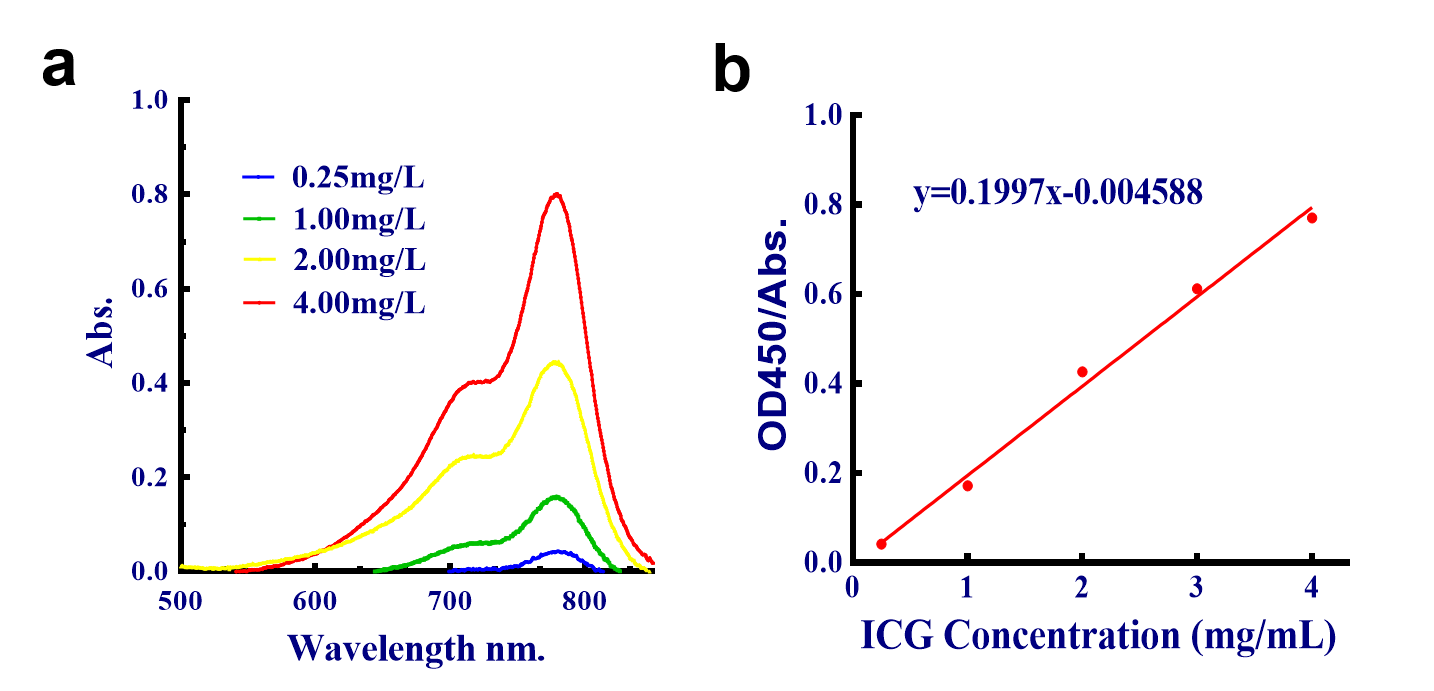


**Supplementary Figure 10.** (a) The UV-Vis absorption spectrum for different concentrations of ICG. (b) The standard curve of ICG.


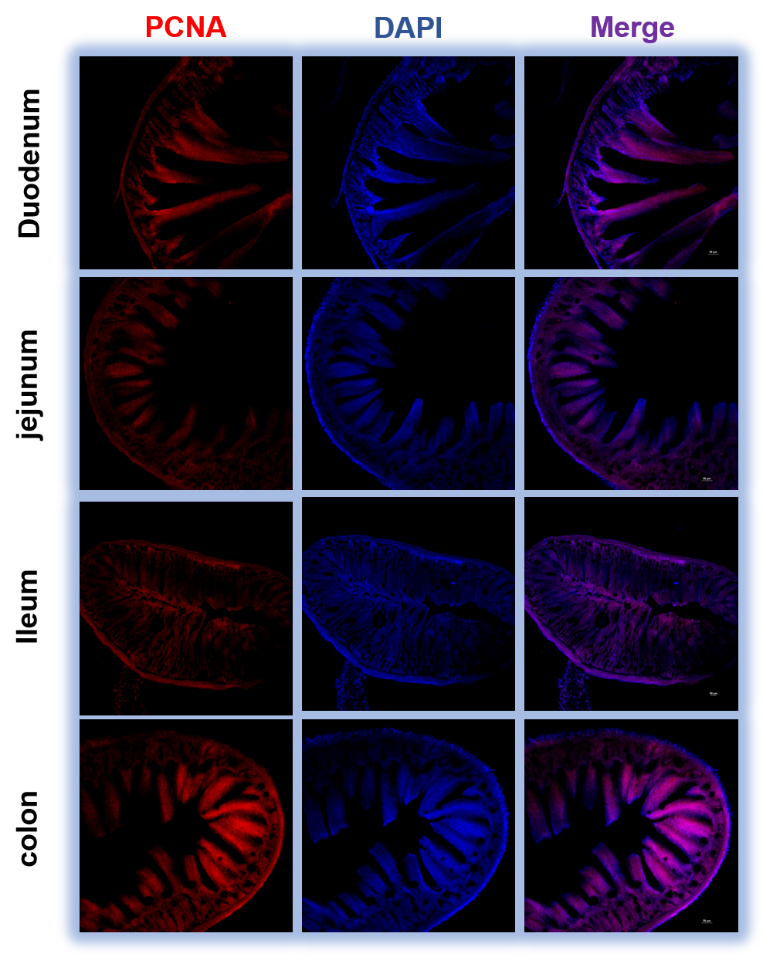


**Supplementary Figure 11.**  Confocal Images of Different Intestinal Segments of Mice——Proliferating Cell Nuclear Antigen (PCNA) Labeling with Fluorescent Signal.


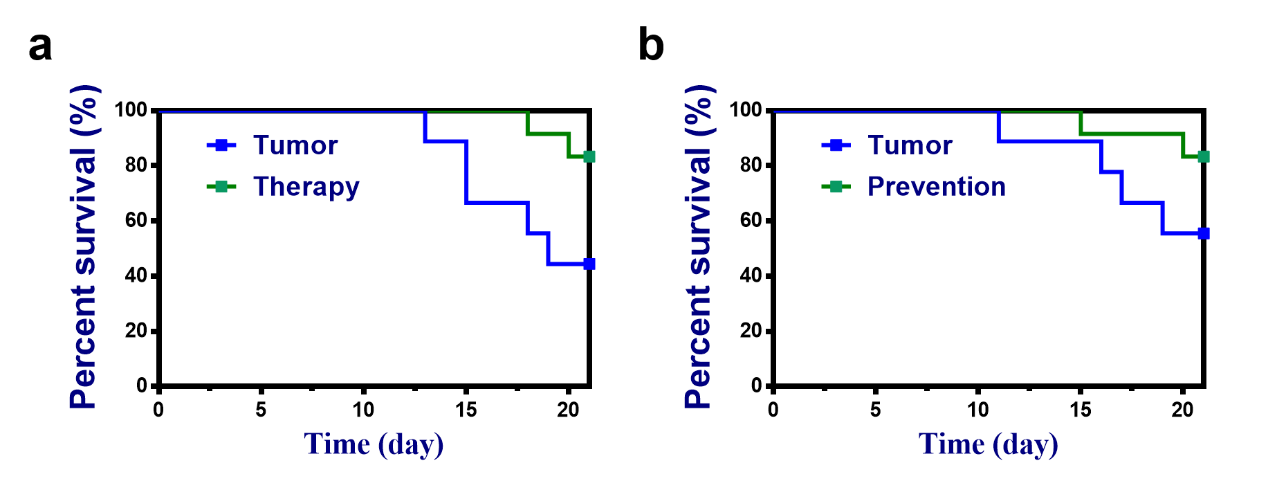


**Supplementary Figure 12.**  Survival curve records of different groups of mice in malignant subcutaneous tumor model. (a) Tumor: Start receiving subcutaneous injection of B16F10 tumor cells on the 1st day. Therapy: Start receiving subcutaneous injection of B16F10 tumor cells on the 1st day. Start receiving treatment of "NIR+UCMs" on the 7th day. (b) Tumor: Start receiving subcutaneous injection of B16F10 tumor cells on the 1st day. Therapy: tart receiving subcutaneous injection of B16F10 tumor cells on the 1st day. Start receiving treatment of "NIR+UCMs" on the 1st day.


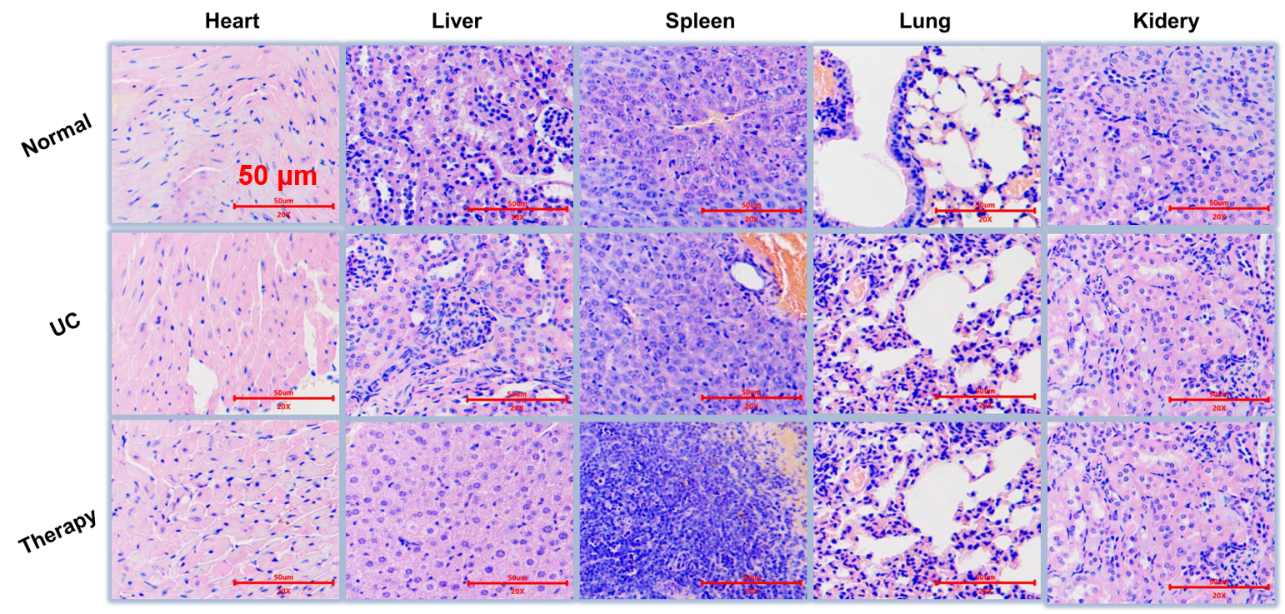


**Supplementary Figure 13.**  Comparison of organ hematoxylin-eosin staining results in different groups of mice in experiments for simulated treatment of ulcerative colitis.


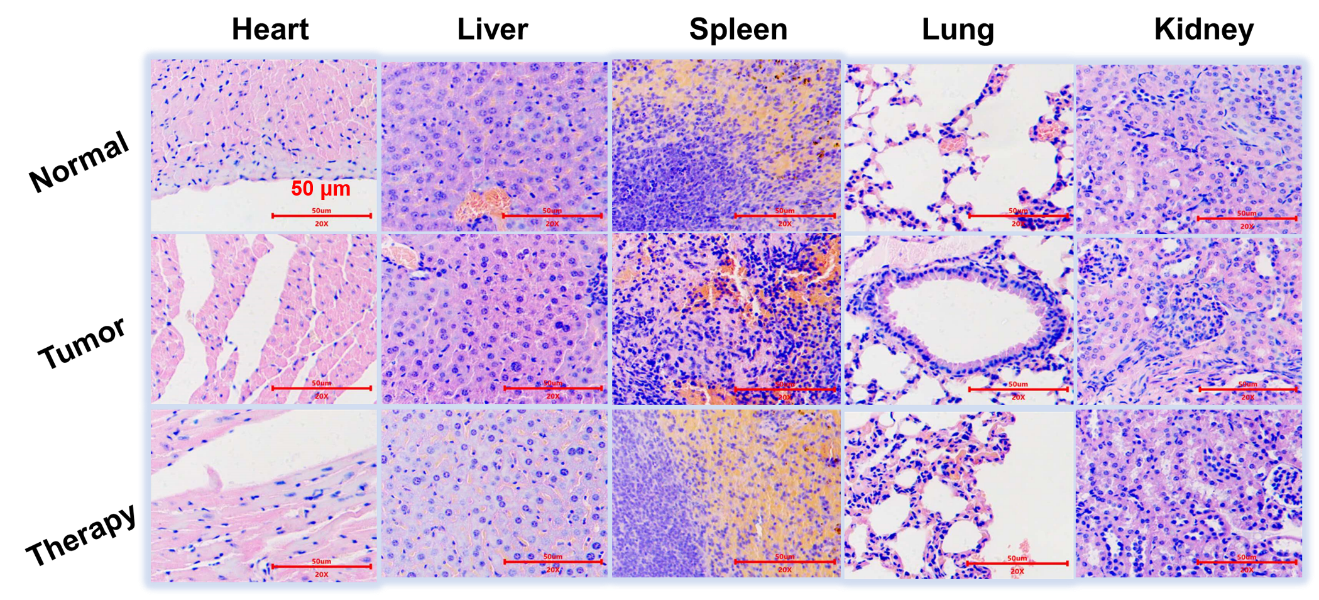


**Supplementary Figure 14.**  Comparison of organ hematoxylin-eosin staining results in different groups of mice in experiments for simulated treatment of anti-melanoma B16F10.


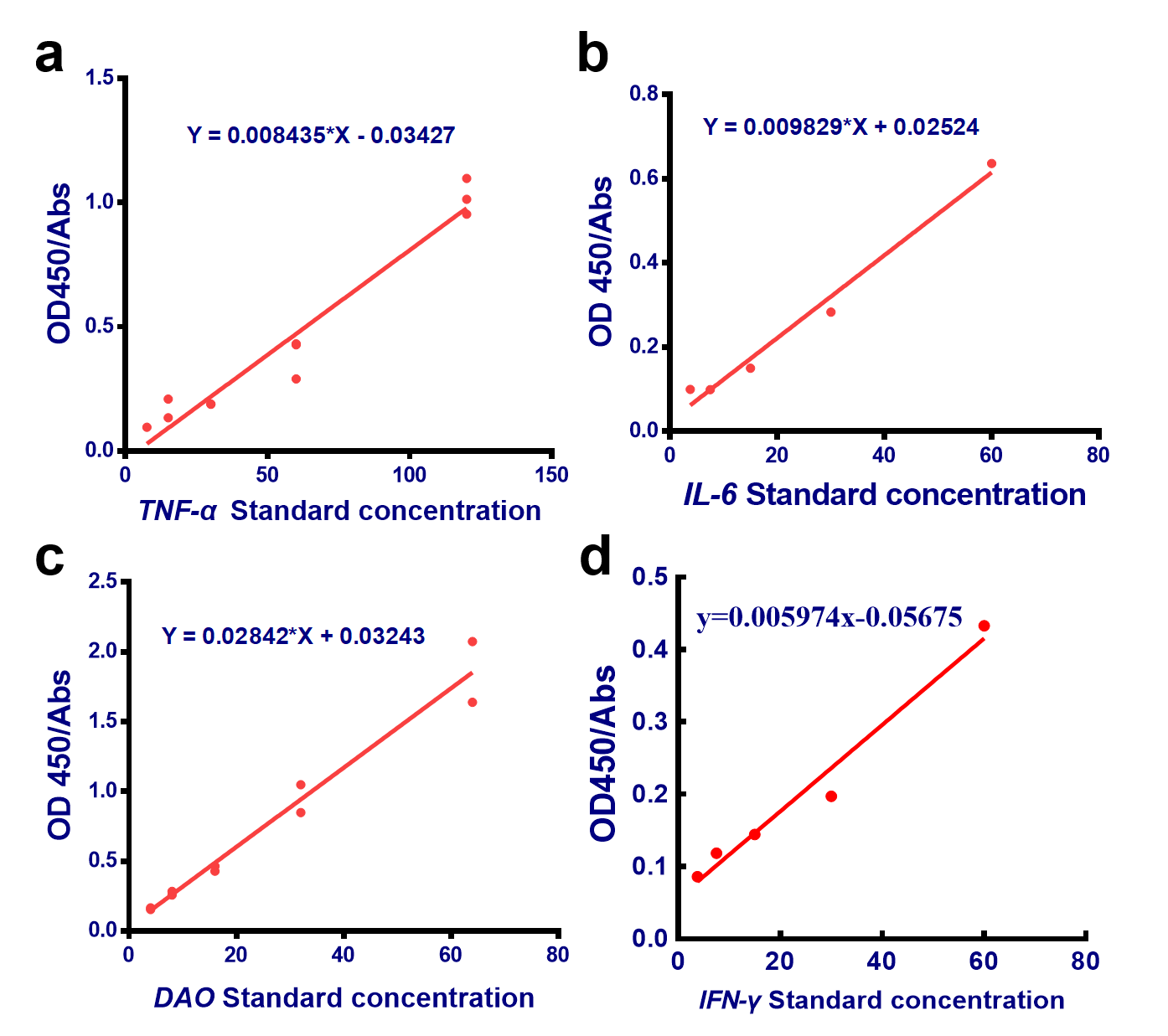


**Supplementary Figure 15.** Determination of Standard Curves of Various Immune Related Factors in ELISA.


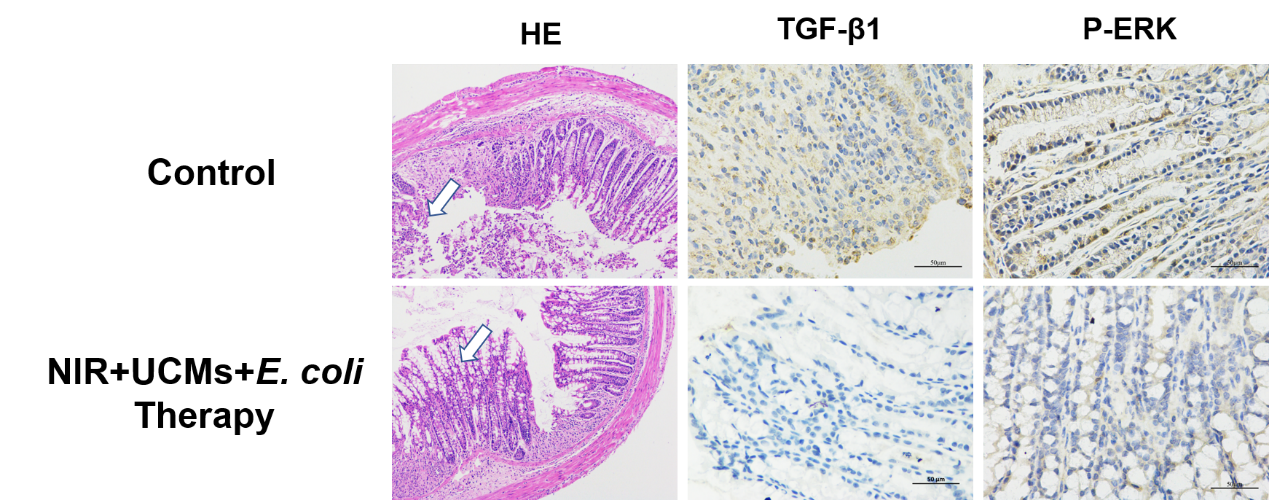


**Supplementary Figure 16.** Analysis of H&E staining and immunofluorescence histochemical staining (TGF-β1 & P-ERK) of case “Therapy”（Figure. 4g）.


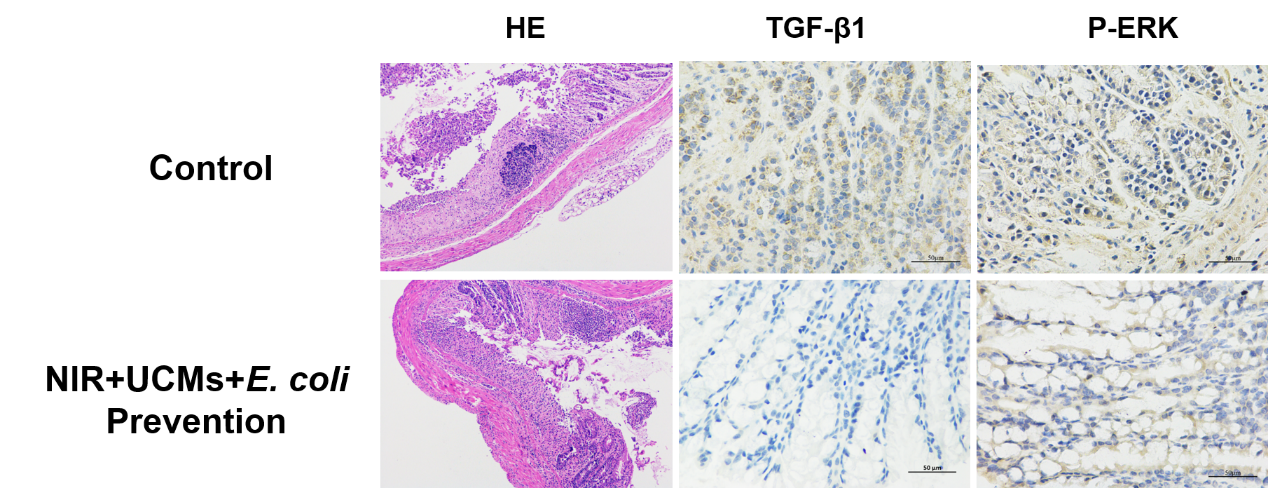


**Supplementary Figure 17.** Analysis of H&E staining and immunofluorescence histochemical staining (TGF-β1 & P-ERK) of case “Prevention”（Figure. 4n）.


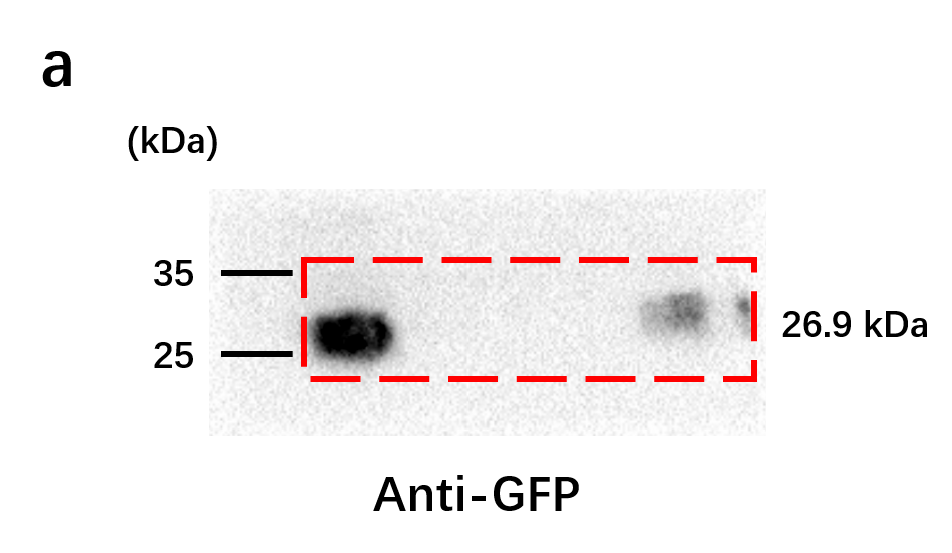

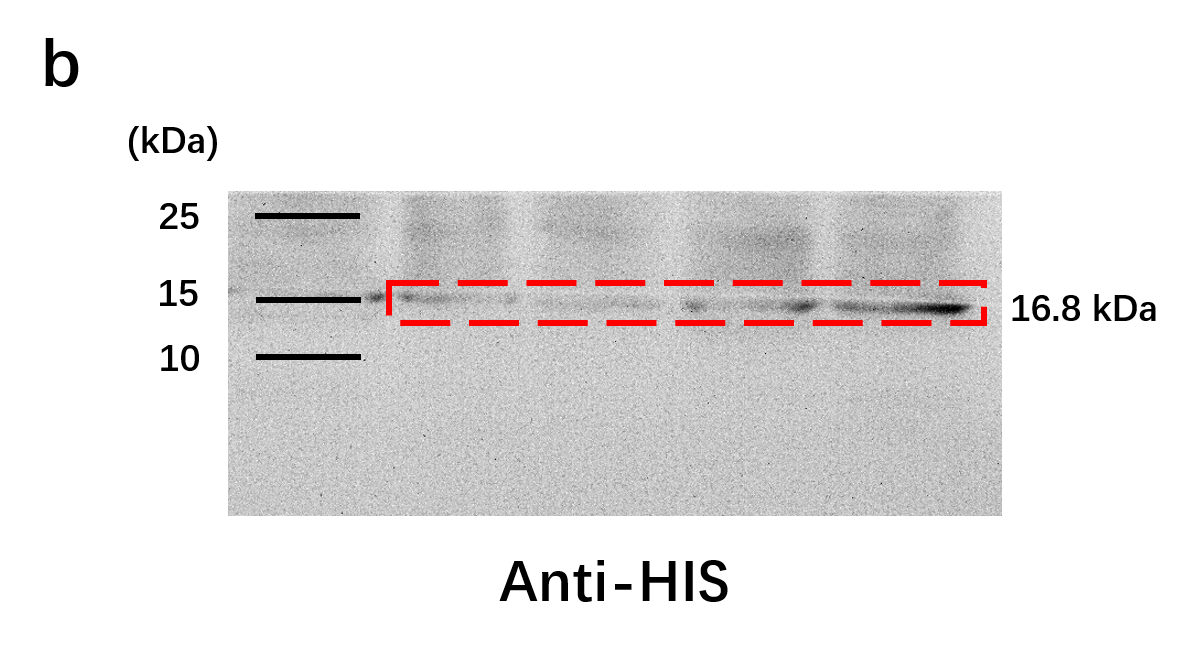


**Supplementary Figure 18.** Uncropped images of gel or blot. Dashed boxes indicate pictures shown in the relevant figure.
