# Supplementary material for: Upconversion optogenetic micro-nanosystem optically controls the secretion of light-responsive bacteria for systemic immunity regulation
Source: Commun Biol. 2020 Oct 9;3:561. doi: 10.1038/s42003-020-01287-4 (PMC7547716; doi:10.1038/s42003-020-01287-4)
Supplement: Supplementary file 2 — Reporting Summary [file 42003_2020_1287_MOESM2_ESM.pdf]

nature research

Corresponding author(s): Hanjie Wang

Last updated by author(s): Aug 22, 2020

Reporting Summary

Nature Research wishes to improve the reproducibility of the work that we publish. This form provides structure for consistency and transparency in reporting. For further information on Nature Research policies, see our Editorial Policies and the Editorial Policy Checklist.

Statistics

For all statistical analyses, confirm that the following items are present in the figure legend, table legend, main text, or Methods section.

n/a	Confirmed	
[ ]	[x]	The exact sample size (*n*) for each experimental group/condition, given as a discrete number and unit of measurement
[ ]	[x]	A statement on whether measurements were taken from distinct samples or whether the same sample was measured repeatedly
[ ]	[x]	The statistical test(s) used AND whether they are one- or two-sided<br>*Only common tests should be described solely by name; describe more complex techniques in the Methods section.*
[x]	[ ]	A description of all covariates tested
[x]	[ ]	A description of any assumptions or corrections, such as tests of normality and adjustment for multiple comparisons
[x]	[ ]	A full description of the statistical parameters including central tendency (e.g. means) or other basic estimates (e.g. regression coefficient) AND variation (e.g. standard deviation) or associated estimates of uncertainty (e.g. confidence intervals)
[ ]	[x]	For null hypothesis testing, the test statistic (e.g. *F*, *t*, *r*) with confidence intervals, effect sizes, degrees of freedom and *P* value noted<br>*Give P values as exact values whenever suitable.*
[x]	[ ]	For Bayesian analysis, information on the choice of priors and Markov chain Monte Carlo settings
[x]	[ ]	For hierarchical and complex designs, identification of the appropriate level for tests and full reporting of outcomes
[x]	[ ]	Estimates of effect sizes (e.g. Cohen's *d*, Pearson's *r*), indicating how they were calculated

Our web collection on statistics for biologists contains articles on many of the points above.

Software and code

Policy information about availability of computer code

Data collection	NIS-Elements C-ER, Flowjo V10, Origin 8.0, GraphPad Prism 6, Image J 1.50i, ChemDraw Professional, Adobe Illustrator 2019.
Data analysis	All statistical analyses were performed on Origin 8.0, GraphPad Prism 6. NIS-ElementsC-ER was used to analyze fluorescent images. Image J 1.50i was used for fluorescence intensity. The molecular structure drawings was drawn by ChemDraw Professional. The editing of the main diagram was done by Adobe Illustrator 2019

For manuscripts utilizing custom algorithms or software that are central to the research but not yet described in published literature, software must be made available to editors and reviewers. We strongly encourage code deposition in a community repository (e.g. GitHub). See the Nature Research guidelines for submitting code & software for further information.

Data

Policy information about availability of data

All manuscripts must include a data availability statement. This statement should provide the following information, where applicable:

- Accession codes, unique identifiers, or web links for publicly available datasets
- A list of figures that have associated raw data
- A description of any restrictions on data availability

The authors declare that all data supporting the results in this study are available within the paper and its Supplementary Information. The datasets generated and analysed during the study are available from the corresponding author upon reasonable request.

Field-specific reporting

Please select the one below that is the best fit for your research. If you are not sure, read the appropriate sections before making your selection.

- [x] Life sciences
- [ ] Behavioural & social sciences
- [ ] Ecological, evolutionary & environmental sciences

For a reference copy of the document with all sections, see nature.com/documents/nr-reporting-summary-flat.pdf

Life sciences study design

All studies must disclose on these points even when the disclosure is negative.

Sample size	Sample sizes were determined according to the results of previous preliminary experiments.
Data exclusions	No data were excluded from the analyses.
Replication	All experimental findings could be reliably reproduced.
Randomization	All samples/organisms were randomly allocated into different experimental groups.
Blinding	Blinding and randomization were applied to all experiments.

Reporting for specific materials, systems and methods

We require information from authors about some types of materials, experimental systems and methods used in many studies. Here, indicate whether each material, system or method listed is relevant to your study. If you are not sure if a list item applies to your research, read the appropriate section before selecting a response.

Materials & experimental systems

n/a	Involved in the study	
x		Antibodies
	x	Eukaryotic cell lines
x		Palaeontology and archaeology
	x	Animals and other organisms
x		Human research participants
x		Clinical data
x		Dual use research of concern

Methods

n/a	Involved in the study	
x		ChIP-seq
	x	Flow cytometry
x		MRI-based neuroimaging

Eukaryotic cell lines

Policy information about cell lines

Cell line source(s)	Mus melanoma cells B16-F10
Authentication	None of the cell line was authenticated.
Mycoplasma contamination	This cell line does not have mycoplasma contamination.
Commonly misidentified lines (See ICLAC register)	No commonly misidentified cell lines were used.

Animals and other organisms

Policy information about studies involving animals; ARRIVE guidelines recommended for reporting animal research

Laboratory animals	C57BL/6 female mouse (6-8 weeks)
Wild animals	The study did not involve wild animals.
Field-collected samples	The study did not involve samples collected from the field.
Ethics oversight	Animal experiments were performed by the statutory requirements of People's Republic of China (GB14925-2010).

Note that full information on the approval of the study protocol must also be provided in the manuscript.

Flow Cytometry

Plots

Confirm that:

☒ The axis labels state the marker and fluorochrome used (e.g. CD4-FITC).

☒ The axis scales are clearly visible. Include numbers along axes only for bottom left plot of group (a 'group' is an analysis of identical markers).

☒ All plots are contour plots with outliers or pseudocolor plots.

☒ A numerical value for number of cells or percentage (with statistics) is provided.

Methodology

Sample preparation	In vitro, the bacterias was collected at 8000rpm for 5min, and washed with PBS twice.<br>In vivo, mice were sacrificed and segment the intestine. The intestinal mucosa was collected and resuspended in PBS at the volume ration of 1:1. Precipitate was removed by centrifugation of 2000rpm for 15min and bacteria was collected by centrifugation of 8000rpm at for 5min. Then the sediment was fixed with 1% PFA for 20min and followed by PBS wash. The sediment was then resuspended in 1ml PBS before flow cytometry analysis. All procedures were kept in low temperature.
Instrument	Flow cytometric analysis was performed using a FACSCalibur instrument (Becton Dickinson, Sunnyvale, CA) equiped with a 488-nm and 561-nm argon laser
Software	Data were analyzed on Flowjo V10
Cell population abundance	No post-sort fractions were collected.
Gating strategy	Cells were first gated on FSC/SSC to select the target colony. GFP gating were then performed on the live cell population.

☒ Tick this box to confirm that a figure exemplifying the gating strategy is provided in the Supplementary Information.
